# *Bacillus subtilis* SOM8 isolated from sesame oil meal for potential probiotic application in inhibiting human enteropathogens

**DOI:** 10.1186/s12866-024-03263-y

**Published:** 2024-03-28

**Authors:** Zhongtian Zhao, Wenrui Li, The Thien Tran, Say Chye Joachim Loo

**Affiliations:** 1 School of Materials Science and Engineering, Nanyang Technological University, Singapore, Singapore; 2 Lee Kong Chian School of Medicine, Nanyang Technological University, Singapore, Singapore; 3 Singapore Centre for Environmental Life Sciences Engineering, Nanyang Technological University, Singapore, Singapore

**Keywords:** *Bacillus subtilis*, Probiotics, Enteropathogen inhibition, Sequencing, Safety

## Abstract

**Background:**

While particular strains within the *Bacillus* species, such as *Bacillus subtilis*, have been commercially utilised as probiotics, it is critical to implement screening assays and evaluate the safety to identify potential *Bacillus* probiotic strains before clinical trials. This is because some *Bacillus* species, including *B. cereus* and *B. anthracis*, can produce toxins that are harmful to humans.

**Results:**

In this study, we implemented a funnel-shaped approach to isolate and evaluate prospective probiotics from homogenised food waste – sesame oil meal (SOM). Of nine isolated strains with antipathogenic properties, *B. subtilis* SOM8 displayed the most promising activities against five listed human enteropathogens and was selected for further comprehensive assessment. *B. subtilis* SOM8 exhibited good tolerance when exposed to adverse stressors including acidity, bile salts, simulated gastric fluid (SGF), simulated intestinal fluid (SIF), and heat treatment. Additionally, *B. subtilis* SOM8 possesses host-associated benefits such as antioxidant and bile salt hydrolase (BSH) activity. Furthermore, *B. subtilis* SOM8 contains only haemolysin toxin genes but has been proved to display partial haemolysis in the test and low cytotoxicity in Caco-2 cell models for in vitro evaluation. Moreover, *B. subtilis* SOM8 intrinsically resists only streptomycin and lacks plasmids or other mobile genetic elements. Bioinformatic analyses also predicted *B. subtilis* SOM8 encodes various bioactives compound like fengycin and lichendicin that could enable further biomedical applications.

**Conclusions:**

Our comprehensive evaluation revealed the substantial potential of *B. subtilis* SOM8 as a probiotic for targeting human enteropathogens, attributable to its exceptional performance across selection assays. Furthermore, our safety assessment, encompassing both phenotypic and genotypic analyses, showed *B. subtilis* SOM8 has a favourable preclinical safety profile, without significant threats to human health. Collectively, these findings highlight the promising prospects of *B. subtilis* SOM8 as a potent probiotic candidate for additional clinical development.

**Supplementary Information:**

The online version contains supplementary material available at 10.1186/s12866-024-03263-y.

## Background

Probiotics are defined, by the Food and Agriculture Organization (FAO) of the United Nations and the World Health Organization (WHO), as viable microorganisms that exhibit a health-promoting effect on the host when ingested in sufficient quantities [1]. Because of their health promoting properties, probiotics have recently attracted significant attention not only among scientists, but also with the general public market including probiotic food and beverages, dietary supplements and animal feed, which estimates project a Compound Annual Growth Rate (CAGR) of 14% from 2023 to 2030 [2]. For a long period, lactic acid bacteria (LAB) such as *Lactobacillus*, *Lactococcus, Streptococcus* and *Bifidobacterium* have been considered to be safe for use [3–5]. Despite the widespread use of various functional LAB in probiotic fermented foods on a global scale, there remains a strong demand within the biofunctional product market for the implementation and expansion of available probiotic products. Therefore, much research effort has focused on the identification and selection of novel strains possessing diverse and distinct functional properties [6]. In fact, novel microbial groups, such as yeast, other strains of LAB and *Bacillus* [7], continue to be discovered by scientists annually [8, 9].

*Bacillus* strains have garnered historical validation for their utility in large-scale enzyme production. They have also been employed as probiotics for human consumption and as direct-fed microbial supplements to enhance animal health over a long period. Their suitability as probiotics stems from their inherent capacity for endospore formation. This characteristic enables them to endure the harsh conditions of low pH and bile salt exposure within the gastrointestinal tracts (GIT) of both humans and monogastric animals [10, 11]. Some strains of *Bacillus*, e.g., *B. coagulans, B. clausii* and *B. subtilis*, have been widely utilised as probiotics in the food and pharmaceutical industry due to this endospore forming property and safe profile [12–14]. *B. subtilis*, in particular, possesses a well-documented history of safe consumption on a global scale. Noteworthy examples include its role in the production of traditional fermented foods such as natto in Japan, kimchi in Korea, and Thua nao in Thailand [15–17]. However, certain *Bacillus* species, including *B. anthracis* and *B. cereus* etc., are known to produce enterotoxins, raising concerns about their safety [18]. In addition, specific *B. subtilis* strains, for example, *B. subtilis* G7 strain obtained from a deep-sea hydrothermal vent exhibits lethality towards vertebrate creatures when deliberately introduced into animals [19]. Therefore, assessing the safety of strains from *Bacillus* are necessary from both phenotypic and genotypic aspects.

Typically, probiotics were discovered and isolated from humans or dairy products such as kefir [20], cheese [21], and fruits [22] as they are perceived as a reliable reservoir of microorganisms, and are considered to be safe and suitable for product development. However, alternative sources such as grains and waste [23] are now being utilised for isolating novel microbe strains. Homogenised food waste, including okara [24], spent coffee grounds [25], spent barley grains [26], and oil pressed cakes [27], offers promising sources for the isolation of specific microbes. These substrates are characterized by their consistent and valuable nutritional profiles, which are conducive to the growth of various microorganisms. Furthermore, the conventional disposal of such food waste in landfills gives rise to significant environmental concerns. The isolation of potential probiotics from these food waste materials can facilitate their reuse in the valorisation of food waste for various potential applications in food [28], animal feed [29], as nutraceuticals [30], and biomedical purposes [31].

## Material and methods

### Materials

The sesame oil meal (SOM) used in this study was sourced from oil processing residues that were generously provided by the Oh Chin Hing sesame oil factory in Singapore. In various assays, we utilised *Lactobacillus plantarum* WT (Wild type strain) and *Lactobacillus rhamnosus* GG (LGG) as positive probiotic controls. The specific human enteropathogens employed in this investigation are detailed in Table 1. All enteric pathogens and human intestinal Caco-2 cell lines (HTB-37™) were purchased from the American Type Culture Collection (ATCC, Manassas, VA, United States) except *Staphylococcus aureus*, which was supplied by our colleagues at the Singapore Centre for Environmental Life Sciences Engineering (SCELSE).

**Table 1:** Human enteropathogens used in this study

Species	Strain	Media	Aerobic/Anaerobic	Temperature (°C)
*Staphylococcus aureus*	USA300	TSB	OX (Aerobic)	37
*Escherichia coli* O157:H7	ATCC43888	TSB	OX	37
*Bacillus cereus*	ATCC 11778	NB	OX	30
*Vibrio parahaemolyticus*	ATCC 17802	NB with 3% w/v NaCl	OX	37
*Salmonella enterica* subsp. Enterica	ATCC-BAA-190	NB	OX	37

De Man, Rogosa, and Sharpe (MRS), Tryptic Soy (TS), Nutrient broth (NB), and Rogosa media were employed for the isolation of strains from SOM. The acquisition of these media was facilitated through Thermo Fisher Scientific (Waltham. MA, United States). Cycloheximide at a concentration of 150 mg/L was added into MRS, NB, TS, and Rogosa media to inhibit yeast growth. The antibiotics were subjected to filtration and subsequently introduced into the respective autoclaved media. Bacto agar was obtained from BD (Franklin Lakes, NJ, United States). In addition, 2,2-diphenyl-1-picrylhydrazyl (DPPH), Dulbecco’s Modified Eagle Medium (DMEM), fetal bovine serum (FBS), and trypsin-ethylenediaminetetraacetic acid (EDTA) were purchased from Thermo Fisher Scientific (Waltham. MA, United States). The Q5 High Fidelity PCR kit was sourced from New England Biolabs (Ipswich, MA, United States), while the CytoTox 96R non-radioactive cytotoxicity kit was obtained from Promega (Madison, WI, United States). The DNeasy Ultraclean Microbial Kit was acquired from QIAgen (Hidden, Germany). All other chemicals used in this study were purchased from Sigma Aldrich (St. Louis, MO, United States). Furthermore, all media except Rogosa, chemical solutions, and apparatus were subject to sterilization through autoclaving at 121 °C for 15 min prior to their utilization.

## Methods

### Isolation of Microbes from SOM

Firstly, the SOM was subjected to aerobic incubation at 37 °C for two days to facilitate a starving approach for the cultivation of microorganisms originally presented in the SOM that can utilise SOM well. Subsequently, the cultured SOM was subjected to a series dilution process using 1 × Phosphate-buffered saline (PBS) solution, wherein 1 mL of the microbe culture was mixed with 9 mL of the PBS solution. Following this, 100 mL of the microbial solution at different concentrations was spread onto different selective agar plates and incubated at 37 °C aerobically for 24 h. MRS and Rogosa media were specifically employed for the isolation of Lactic acid bacteria (LAB), as the majority of known or commercially available probiotic strains belong to this group. NB and TS media were employed as general broths.

After 24 h of incubation, individual colony of different microbes was selected based on their characteristics such as form (circular, filamentous, etc.), elevation (raised, flat, etc.), margin (filiform, lobate, etc.), surface (smooth, rough, etc.), opacity (transparent, opaque, etc.), and pigmentation (white, purple, etc.) [32]. A single colony of each microbe was then be inoculated into the corresponding broth, followed by another day of incubation at 37 °C aerobically. Subsequently, the incubated microbial cultures were streaked onto agar plates once again for purification. Finally, the single colony of each microbe was inoculated and preserved in a -80 °C freezer for further study. The stock solution was prepared by combining 900 mL of the microbial culture with 300 mL of a 60% (v/v) glycerol solution, resulting in a total glycerol concentration of 15% (v/v).

### Identification of Isolated Species by 16S rRNA

The 16S rRNA was sequenced to determine the species identity of SOM derived microbial strains. Genomic DNA was isolated from respective microbial culture using DNeasy Ultraclean Microbial Kit in accordance with the manufacturer’s instructions. The Q5 High Fidelity PCR kit was used with universal primers 27F and 1492R to amplify 16S rRNA for bacteria. The PCR reaction mix consisted of 10 µL 5X Q5 reaction buffer, 1 µL 10 mM dNTPs, 2.5 µL 10 mM forward primer, 2.5 µL 10 mM reverse primer, 0.5 µL Q5 High Fidelity DNA polymerase, 5 µL DNA template, and 28.5 µL nuclease-free water, total 50 µL. PCR amplification was carried out with the following parameters: 98 °C for 3 min, 30 cycles (98 °C for 10 s, 55 °C for 15 s, 72 °C for 90 s), 72 °C for 2 min, and holding at 4 °C. PCR products were checked by gel electrophoresis using the Gel Doc system (Bio-Rad Laboratories, Hercules, CA, United States). PCR products at the predicted size were sent to an external vendor (1st base, Singapore) for sequencing. Obtained nucleotide sequences were analysed using the ApE plasmid editor software [33], and species assignment of SOM isolates was done using the National Centre for Biotechnology Information (NCBI) BLAST platform, based on the BLAST result which yielded the highest total score.

### Agar well diffusion assay to assess antipathogenic activity

The experimental procedure followed the protocol proposed by Tan et al. with modifications [34]. Five human enteropathogens listed in Table 1 were inoculated into respective broth to grow for 24 h. Then pathogen cultures were appropriately diluted to an initial OD600 of 0.1 in their respective media. Subsequently, 100 µL of the diluted pathogen cultures were spread onto agar. To create wells, 6 mm-diameter cavities were carefully made and these wells were subsequently filled with 50 µL of microbial cultures containing isolated microbes from SOM. Following the preparation of the plates, they were incubated under growth conditions specific to the pathogens for 24 h. Finally, the plates were examined for the presence of inhibition zones surrounding individual wells. These inhibition zones were characterized by clear areas devoid of visible pathogen growth. Inhibition zones measuring greater than 4 mm, ranging between 2 and 4 mm, and less than 2 mm were classified as strong (+ + +), intermediate (+ +), and weak inhibition ( +), respectively [34].

### Whole genome sequencing for genotypic characterization

The selected potential probiotics *B. subtilis* SOM8 (after phenotype screening and 16S rRNA) underwent further genotypic characterization through WGS. Genomic DNA was extracted from these isolates using the DNeasy Ultraclean Microbial Kit following the manufacturer's instructions. Prior to sequencing, the quality and concentration of the extracted DNA were assessed through gel electrophoresis and a Qubit 2.0 Fluorometer, respectively. Then the DNA samples were sent to an external vendor (Azenta Life Sciences, Singapore). The quality of raw reads was verified using FastQC [35] and the quality of the assembled contigs was assessed using the DDBJ Fast Annotation and Submission Tool (DFAST). 

The assembled contigs of the chosen *B. subtilis* SOM8 strain with most promising antipathogenic activities were submitted to GenBank under the BioProject ID PRJNA1009692, with accession number JAVICJ000000000. Functional gene annotation of the assembled contigs was performed using the NCBI prokaryotic genome annotation pipeline. Second metabolites, bacteriocins, virulence factors, and antimicrobial resistance (AMR) genes were identified using the antiSMASH [36], BAGEL4 [37], Virulence factors Database (VFDB) [38] and Comprehensive Antibiotic Resistance Database (CARD) [39] respectively. Plasmids and Mobile Genetic Elements (MGEs) were identified using PlasmidFinder 2.1 [40] and MobileElementFinder [41].

Taxonomic analysis was conducted using the Type Strain Genome Server (TYGS) [42]. Specifically, the genomes of isolates were compared against all type strain genomes present in the TYGS database to identify closely related type strains. These strains were then compared pairwise to determine their intergenomic distances, which were subsequently used to construct a balanced minimum evolution tree with branch support through FASTME 2.1.6.1 [43].

### Minimum Inhibitory Concentration (MIC) Evaluation

The MIC protocol strictly followed Clinical and Laboratory Science Institute (CLSI) M07 standard [44] and European Food Safety Authority (EFSA) MIC Resistance Threshold for *Bacillus* strains [45]. In general, eight commonly prescribed antibiotics (chloramphenicol, clindamycin, erythromycin, gentamicin, kanamycin, streptomycin, oxytetracycline, and vancomycin) were used for MIC evaluation of isolated *B. subtilis* SOM8, as can be seen in Supplementary Figure S1.

In general, a 96-well microplate was utilised for the experiment. To each well, 100 µL of different kinds of drugs were introduced into Well 1, and from Well 2 to Well 12, 50 µL of broth without bacteria was dispensed. Subsequent to this, a sequential process was followed: 50 µL of the drug solution from Well 1 was transferred until Well 10. At this point, 50 µL of the resultant mixture was extracted from Well 10 and discarded, resulting in uniform 50 µL solutions across all wells. For all antibiotics except streptomycin, the initial concentration added was 64 µg/mL, resulting in a concentration of 0.125 µg/mL in well 10. For streptomycin, the initial concentration was set at 1024 µg/mL, leading to a concentration of 2 µg/mL in the well. Following this, 50 µL of a bacterial culture was added, starting from Well 1 up to Well 11, while Well 12 received 50 µL of broth without bacteria, serving as a negative control.

The bacterial culture added to the wells was standardized to a concentration of 10^6^ colony forming units (CFU) CFU/mL, in accordance with CMSI standards, to achieve a consistent final concentration of 5 × 10^5^ CFU/mL. This precise standardization is crucial because the initial bacterial concentration significantly affects MIC results. After standardization, the microplate underwent an incubation period of 16 to 20 h at 37 °C.

### Acid and bile resistance

The experimental procedure for this assay is based on the methodology described by Tan et al. [10] and aims to assess the survivability of isolated microbes under 2 h exposure to acid and bile salts. For this purpose, broths were adjusted to pH 2, 3, and 4 and/or supplemented with 0.5%, 1.0%, and 1.5% (w/v) ox-bile. The culture of microbes was prepared by incubating inoculated microbes for 24 h. Subsequently, 100 µL of the microbial culture was inoculated into 4.9 mL of each respective broth medium, followed by incubation at 37 °C for 2 h under continuous shaking conditions (200 rpm). Enumeration of CFUs was performed through drop plating (100 µL solution, spread onto the agar plates, incubation for 1–2 days) before and after exposure to acid/bile treatment.

### Simulated Gastric Fluids/Simulated Intestinal Fluids (SGF/SIF) Resistance

The experimental procedure for this assay is based on the methodology described by Tan et al. [34, 46] and aims to evaluate the survival of isolated microbes in the human GIT environment by subjecting them to SGF and SIF. The SGF was prepared as a solution of 0.2 M NaCl, 2000 units/mL porcine pepsin, with a pH of 2 using HCl. The SIF was prepared by combining PBS with a pH of 7.4 and 0.3% ox-bile salts and 0.1% pancreatin [47]. 100 µL of the culture of isolated microbes were inoculated into 4.9 mL SGF or SIF, followed by incubation at 37 °C for 2 h under continuous shaking conditions (200 rpm). Enumeration of CFUs was performed before and after exposure to SGF or SIF.

### Heat stability

The experimental protocol for this assay is based on the methodology outlined by Feng et al. [48]. The objective of this study is to evaluate the viability of isolated microorganisms under conditions of elevated temperature, thus providing valuable insights for subsequent industrial processing techniques, including spray drying. The microbial culture tubes were subjected to incubation in a water bath at temperatures of 40, 60, and 80 °C for 30 min. Enumeration of CFUs was conducted before and after exposure to varying temperatures.

### Antioxidant Activity (DPPH assay)

The experimental procedure follows the DPPH scavenging protocol proposed by Luang-In V. and Deeseenthum S. [49] with modifications: Microbial cultures (0.5 mL) were combined with 0.05 mM DPPH in absolute ethanol (3 mL) in duplicate. Controls were prepared by mixing broth with absolute ethanol (3 mL). Subsequently, the reaction mixture was incubated in darkness at room temperature for 30 min. The presence of antioxidant activity was indicated by a discernible colour transition from deep violet to light yellow. Following incubation, the solution was centrifuged at 8000 g$\times$ for 10 min to spin down substances. Then the absorbance at 517 nm was quantified using a spectrophotometer. The antioxidant activity percentage (AA%) was determined using the following ***Eq. ***1. L. Ascorbic acid was used as positive control, *L. plantarum*, a common commercial probiotic was used for comparison.

1 $$AA\mathrm{\% }= [1 - ({A}_{sample} / {A}_{control})]$$

Here, A_sample_ represents the average absorbance at 517 nm measured for the sample with DPPH added (A: 0.5 mL culture + 3 mL DPPH ethanol solution), subtracted by the absorbance of broth without DPPH added (B: 0.5 mL broth + 3 mL absolute ethanol), while A_control_ denotes the absorbance at 517 nm measured for broth with DPPH added (C: 0.5 mL broth + 3 mL DPPH ethanol solution), minus the absorbance of broth without DPPH added. The ***Eq. ***1 can be simplified as below.

$$AA\% = \left[1 - \frac{A-B}{C-B}\right]=\frac{C-A}{C-B}$$

### BSH (Bile salt hydrolase) Activity Assay

The experimental procedure follows Tan et al. [34] with modifications. A volume of 5 µL of the isolated microbial culture was dispensed onto two sets of TS agar plates: one set containing 0.5% (w/v) taurodeoxycholate hydrate (TDC) and the other set without TDC supplementation. The plates were then incubated at 37 °C for 24 h. The presence of BSH activity was assessed by the appearance of a distinctive white precipitate, which corresponds to the deconjugated bile acid on the TDC-supplemented agar plates after 48 h incubation. *L. plantarum* WT was used as positive control.

### Haemolytic activity

Isolated strains were subjected to haemolysis testing on Columbia agar supplemented with 5% (v/v) sheep blood. This was achieved by streaking bacterial cultures on blood agar plates, followed by incubation at 37 °C under aerobic conditions for 24–48 h. The haemolytic activity of the isolates was determined based on the presence of a clear or green halo around the bacterial colonies. Bacterial strains exhibiting a clear halo were categorized as β-haemolytic (complete lysis of red cells, such as *S. agalactiae, S. aureus*), while those with a green halo were considered α-haemolytic (partial or green haemolysis associated with reduction of red cell haemoglobin, such as *S. pneumonia*). Isolates without any halo surrounding the colonies were designated as γ-haemolytic (slight or nonhaemolytic, such as *Enterococcus faecalis*) [50] as can be seen in Supplementary Figure S2.

### Cell Cytotoxicity Using Caco-2 cells (CCK-8 Assay)

The cytotoxicity of the isolated microbial culture was assessed using Caco-2 cells, employing the CytoTox 96 non-radioactive cytotoxicity kit: Cell Counting Kit – 8 (CCK-8). Caco-2 cells were revived and cultured in DMEM supplemented with 10% (v/v) FBS and 1% Non-Essential Amino Acids (NEAA) and maintained at 37 °C in a humidified atmosphere with 5% CO_2_ for seven days to form a confluent monolayer. For the cytotoxicity assay, 100 µL of Caco-2 cell suspensions were seeded into each well of a 96-well microplate (5000 cells/well). The microplate was then incubated overnight to allow cells to adhere to the wells. Subsequently, the medium in each well was replaced with 100 µL of different concentrations of cell-free filtrate (0.005, 0.05, 0.5, 5, 50, 500 µL cell-free filtrate/mL completed DMEM medium, prepared by filtering 24 h fermented bacterial culture using 0.22 μm filter) and 100 µL of different concentrations of lyophilized cell-free filtrate dissolved in DMEM (0.1, 1, 10, 100, 1000, 10,000 μg/mL completed DMEM medium, prepared by filtering 24 h fermented bacterial culture using 0.22 μm filter and freeze-dried). After incubation for 24 h, 10 µL of CCK-8 solution was added to each well and incubated at 37 °C for an additional 4 h. The background absorbance was determined using 100 µL completed DMEM medium and completed DMEM medium with different concentrations of (lyophilized) cell-free filtrate without Caco-2 cells (Cell-free filtrate itself has colour, which will affect the absorbance), while the OD_untreated_ group was prepared by incubating Caco-2 cells using 100 µL PBS solution (Negative control). OD_treated_ was determined with 100 µL complete DMEM medium with Caco-2 cells (Positive control) or different filtrate treatment with Caco-2 cells. The commercial probiotic strain LGG and *B. subtilis* ATCC 6051 were employed for comparison.

Cytotoxicity effect was measured using a spectrophotometer at 450 nm.

$$\mathrm{Cell viability}=\frac{{{\text{OD}}}_{{\text{treated}}}}{{{\text{OD}}}_{{\text{untreated}}}}\times 100\mathrm{\%}$$

### Adhesion Capacity Assay Using Caco-2 Cells

The adhesion capability of microbial cultures to an intestinal surface was evaluated through an in vitro adhesion assay employing the human epithelial cell line Caco-2, following the methodology outlined by Ayala et al. [51]. The assay entails seeding Caco-2 cells at a density of 2.8 × 10^4^ cells/cm^***2***^ in 12-well tissue culture plates, with the culture medium being refreshed daily for 21 days to facilitate growth until the late post-confluence stage. During the final medium change, DMEM without antibiotics is employed.

Subsequently, duplicate confluent monolayers of Caco-2 cells were inoculated with 1 mL of the microbial culture, adjusted to a concentration of 10^**8**^, 10^**7**^, and 10^6^ CFU/mL. Before inoculation, the microbial culture underwent washing with PBS solution and was subsequently resuspended in DMEM. The inoculated Caco-2 plates were then incubated for 2 h under controlled conditions of 37 °C and 5% CO_2_ to facilitate microbial attachment. After incubation, non-attached or loosely adherent microbes were removed by performing three washes of the Caco-2 monolayers using sterile PBS.

For the detachment of adherent microbes, 200 mL of a trypsin solution with a concentration of 0.25% (w/v) and supplemented with 0.53 mM EDTA were added to each well, followed by a 10-min incubation at 37 °C and 5% CO_2_. Subsequently, PBS (800 mL) was pipetted into each well to dilute the trypsin–EDTA solution, and ten-fold serial dilutions were prepared. Drop-plating was conducted to enumerate the CFU of the attached isolated microbes. The percentage of adhesion was calculated by dividing the number of attached microbes by the initial CFU count of the added microbes.

### Statistical analysis

All data were presented as the mean ± standard deviation (SD). Statistical analyses were performed using GraphPad Prism 9 software. One-way and/or two-way ANOVA was employed for comparisons among various groups, and t-test was used to assess differences between two groups. Significance levels were denoted as follows: *, *p* < 0.05; **, *p* < 0.01; ***, *p* < 0.001; ****, *p* < 0.0001, indicating statistical significance.

## Results

### Isolated Strains from SOM

A total of 23 distinct strains were isolated from SOM based on the isolation process. Among these, nine strains demonstrated notable antipathogenic properties, effectively inhibiting the growth of the listed human enteropathogens, as shown in Supplementary Figure S3.

### Antipathogenic Activities (Agar Well Diffusion Assay)

Out of the 23 strains isolated from SOM, nine isolates exhibited notable inhibitory activities against common human enteropathogens. The results of the inhibitory activities of these nine strains are presented in Table 2. The raw data on the diameters of the inhibition zones are provided in Supplementary Table S1.

**Table 2:** Isolated strains’ inhibition to human enteropathogens

Species	Strain	Pathogens				
		***S. aureus***	***E. coli O157:H7***	***B. cereus***	***S. enterica***	***V. parahaemolyticus***
***B. subtilis***	**1**	+ -	+ + + +	+ +	+ +	+ -
	**2**	+ + + + +	+ +	+ + + +	+ -	- +
	**3**	+ + + + + +	+ + + + +	+ + + + + +	+ + + +	+ + + +
	**4**	+ + + + +	+ + + +	+ + + +	+ +	+ + + +
	**5**	+ +	+ + + +	+ + + +	+ +	+ + + + +
	**6**	+ + + +	+ + + + +	+ + + + + +	+ + + +	+ + + +
	**7**	+ + + + +	+ + + +	+ + + + +	+ +	+ + + + + +
	**8**	+ + + + + +	+ + + + +	+ + + + + +	+ + + +	+ + + + + +
***Weissella paramesenteroides***	**1**	- -	+ +	+ + + + +	+ +	+ + + + + +

Each assay was conducted in duplicate, and inhibition zones were categorized as follows: Zones of inhibition exceeding 4 mm were categorized as strong inhibition (+ + +), those measuring between 2 and 4 mm were considered intermediate inhibition (+ +), while zones smaller than 2 mm were regarded as weak inhibition ( +), with (-) signifying the absence of any discernible zone of inhibition [34]

Within this group of nine strains, eight were identified via 16S rRNA sequencing as members of the *B. subtilis* species, with the remaining one strain classified as *Weissella paramesenteroides*. Among these strains, *B. subtilis* SOM8 displayed the most promising inhibitory activities against all five selected human enteropathogens. Notably, *B. subtilis* SOM8 exhibited remarkable inhibitory effects on the growth of common foodborne pathogens, including *V. parahaemolyticus* and *B. cereus*, which can cause diarrhoeal diseases, as well as the virulent serotype *E. coli* O157:H7, responsible for diarrhoea and associated complications. Furthermore, *B. subtilis* SOM8 exhibited inhibitory effects on not only Gram-positive pathogens (*S. aureus,* and *B. cereus*) but also Gram-negative pathogens (*E. coli*, *S. enterica*, and *V. parahaemolyticus*). Consequently, *B. subtilis* SOM8 was selected for further comprehensive phenotypic and genotypic screening.

### Taxonomic Information of B. subtilis SOM8

**Fig. 1:**
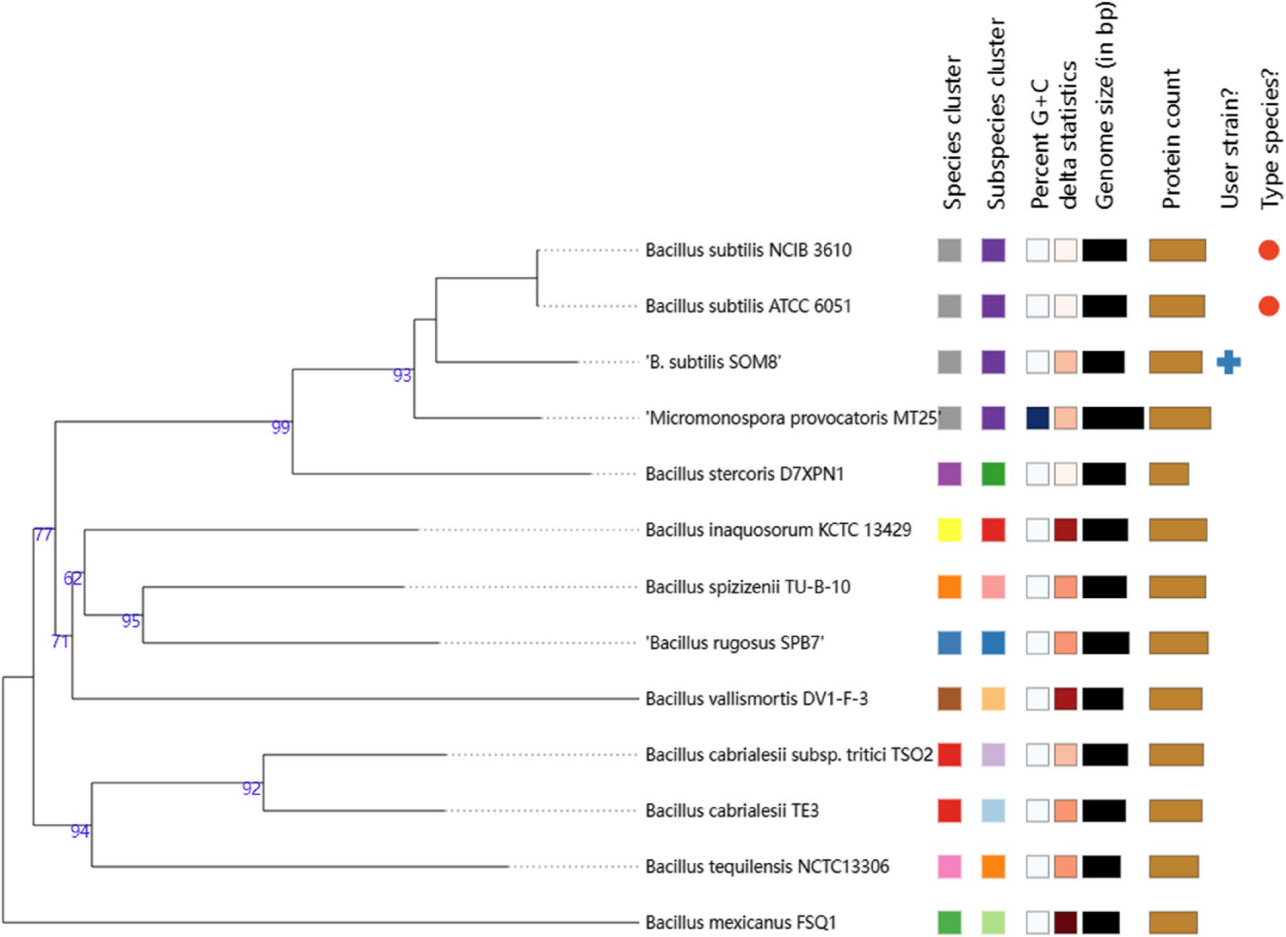
Phylogenetic tree of B. subtilis SOM8 with similar Bacillus strains using TYGS database

Phylogenetic analysis was employed to discover the relationship between *B. subtilis* SOM8 and several closely related strains. A phylogenetic tree of *B. subtilis* SOM8 was constructed using TYGS, as illustrated in Fig. 1, the raw data of TYGS results are shown in Supplementary Table S2 and S3. *B. subtilis* SOM8 was found to share close phylogenetic proximity with well-known wild-type strains, notably *B. subtilis* NCIB 3610 and *B. subtilis* ATCC 6051. Moreover, in alignment with the BLAST results in prior research, *B. subtilis* SOM8 also demonstrated a close taxonomic alignment with *B. subtilis* subsp. subtilis 168, as well as a commercially available probiotic strain, *B. subtilis* MB40 [10]. These findings underscore the substantial potential of isolated *B. subtilis* SOM8 for application as a probiotic.

### Acid and bile tolerance

**Fig. 2:**
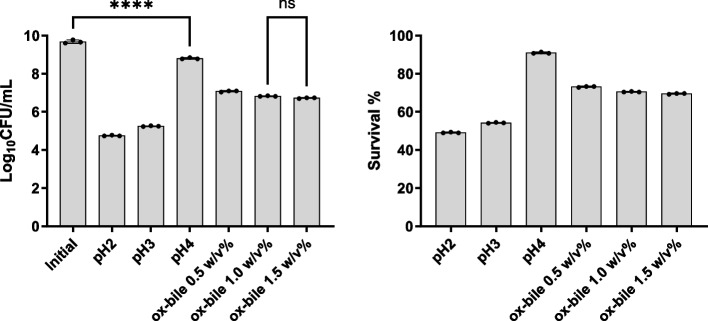
B. subtilis SOM8 tolerance to acid and bile salt. Log_10_CFU/mL of B. subtilis SOM8 was measured before and after exposure to acid or ox-bile salts for 2 h, respectively

**Fig. 3:**
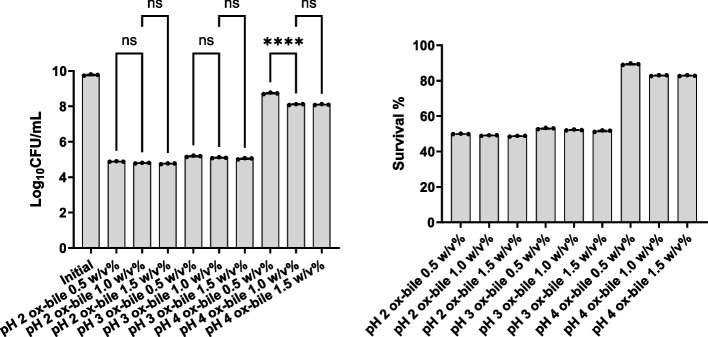
B. subtilis SOM8 tolerance to mixed acid and bile salts conditions. Log_10_CFU/mL of B. subtilis SOM8 was measured before and after exposure to acid with ox-bile salts for 2 h, respectively

The results depicting survival of *B. subtilis* SOM8 after exposure to acid (pH 2, 3, and 4) and varying concentrations of ox-bile salts (0.5%, 1.0%, 1.5% w/v), as well as mixed acid and bile salt conditions for 2 h, are presented in Figs. 2 and 3. Notably, *B. subtilis* SOM8 exhibited susceptibility to low pH conditions (pH 2 and pH 3), resulting in a reduction of 4 to 5 Log_10_CFU/mL. However, under pH 4, the reduction in Log_10_CFU/mL was less than 1, indicating a good survivability to acidic environments when the pH exceeded 4. In the context of bile salts, *B. subtilis* SOM8 demonstrated Log_10_CFU/mL reductions ranging between 2 and 3 across various concentrations, indicating its great tolerance to bile salts. Furthermore, it is noteworthy that under both pH 4 and bile salt conditions, the final Log_10_CFU/mL count for *B. subtilis* SOM8 remained consistently above 6. This observation suggests the considerable potential of *B. subtilis* SOM8 to establish a colony within the human GIT, thereby contributing to its functional role [52]. In addition, when *B. subtilis* SOM8 was exposed to mixed acid and bile salt conditions, the survival pattern was closely related to that observed under solely acidic stress conditions, emphasizing the dominant role of acid in affecting the survivability of *B. subtilis* SOM8.

### SGF/SIF Tolerance

**Fig. 4:**
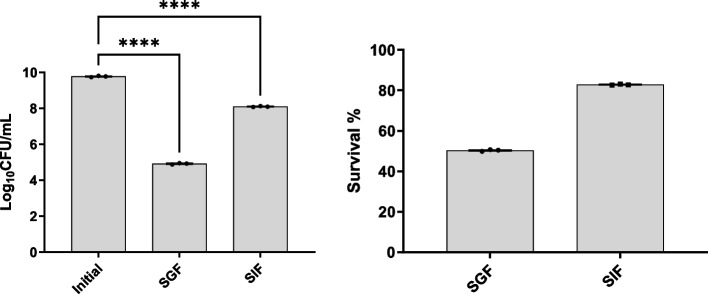
B. subtilis SOM8 tolerance to SGF and SIF, respectively

The susceptibility of *B. subtilis* SOM8 to SGF and SIF was assessed to simulate the gastrointestinal conditions, with the results presented in Fig. 4. *B. subtilis* SOM8 demonstrated robust survival in SIF, maintaining a Log_10_CFU/mL count exceeding 8 after 2 h. Conversely, the strain exhibited relative susceptibility to SGF due to its low acidic environment (pH 2) and the presence of porcine pepsin, resulting in a reduction of Log_10_CFU/mL between 4 and 5. Nonetheless, the survivability remained at approximately 50%, signifying a better tolerance to both SGF and SIF than mostly applied commercial probiotic strain LGG [53]. Therefore, *B. subtilis* SOM8 exhibits substantial potential for applications as probiotics, with the possibility of encapsulation to enhance its survivability under human GIT.

### Heat stability

**Fig. 5:**
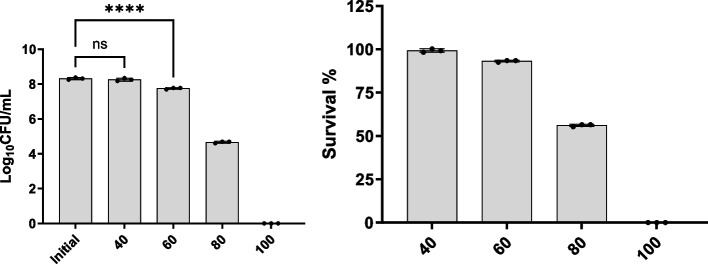
B. subtilis SOM8 tolerance to elevated temperatures from 40 °C to 100 °C for 30 min

The outcomes of this investigation are presented in Fig. 5. Evidently, the figure depicts that the amount of the bacteria remained constant under 40 °C, with Log_10_CFU/mL reduction smaller than 0.1. As the temperature elevated to 60 °C, a minor decline in the Log_10_CFU/mL from 8.3 to 7.8 was observed, with survivability retained at more than 90%. However, a substantial decline was observed as the temperature reached 80 °C, leading to a reduction in the Log_10_CFU/mL to 4.7. Notably, the exposure of the bacteria to 100 °C for the same duration resulted in complete cell inactivation.

### Antioxidant Activity (DPPH Scavenging Assay)

**Fig. 6:**
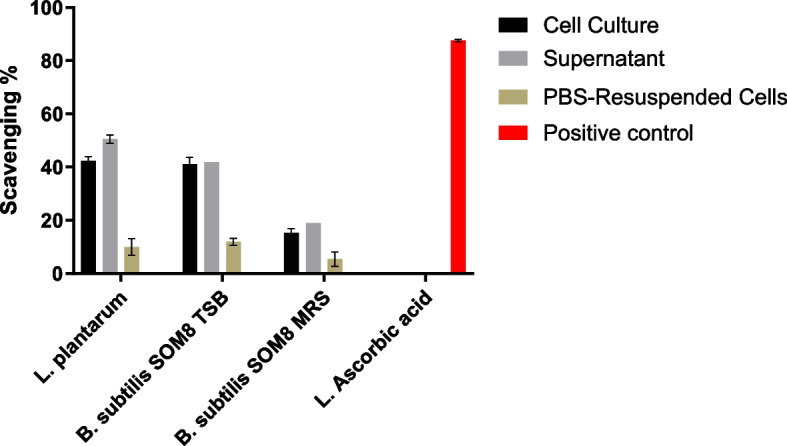
Antioxidant activity of B. subtilis SOM8 in TS, MRS broth, respectively

**Fig. 7:**
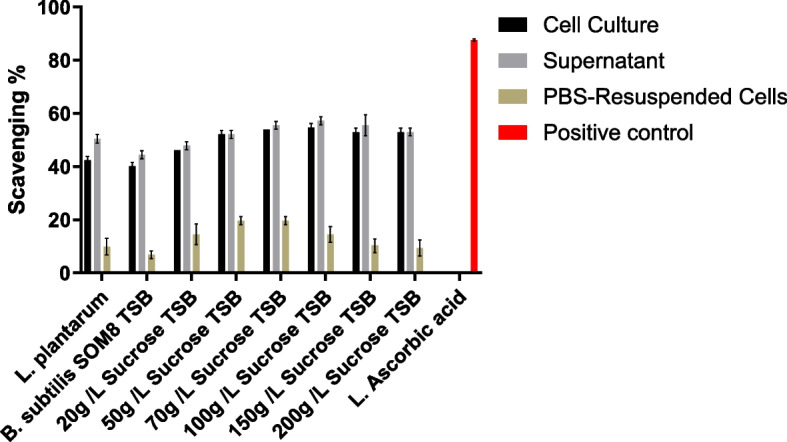
Antioxidant activity of B. subtilis SOM8 in TS broth with different concentrations of supplemented sucrose

The antioxidant activities of the cell culture, supernatant, and PBS-resuspended cells of *B. subtilis* SOM8 are illustrated in Figs. 6 and  7, respectively. In Fig. 6, it reveals that *B. subtilis* SOM8 in TS broth, exhibited a remarkable antioxidant activity, evidenced by an approximate 40% DPPH scavenging, similar to that of *L. plantarum*. Contrarily, *B. subtilis* SOM8 in MRS broth resulted in low antioxidant activity, with DPPH scavenging ranging between 10 and 15%. Notably, all PBS-Resuspended cells including dissoluble metabolites displayed relatively lower antioxidant activity, while the original cell culture and the supernatant exhibited comparatively high antioxidant activity. This discrepancy could be attributed to the fact that the antioxidant activity primarily originates from secondary metabolites present in the supernatant, such as the exopolysaccharide (EPS) and organic acids produced by the cells. When *B. subtilis* SOM8 is introduced into MRS, a broth with relatively lower pH that is usually used for growing lactic acid bacteria, it appears to augment biofilm production [54]. Such a strategy is usually employed by microbes to overcome harsh environments like low pH, rather than synthesizing secondary metabolites for antioxidant activity. In Fig. 7, the data illustrates a progressive enhancement in antioxidant activity, reflected by the DPPH scavenging increasing from 40 to 60%, as supplementary sucrose is incrementally introduced into the TS broth, up to a concentration of 150 g/L. Therefore, there exists a saturation point, beyond which adding more sucrose will have no effect on antioxidant activity. The results also proved the dominant role of sucrose instead of monosaccharide in producing EPS [55].

### BSH activity

The BSH activity of isolated *B. subtilis* strains SOM 1–8 were shown in Supplementary Figure S4. Evidently, all eight strains of isolated *B. subtilis* exhibited BSH activities, as signified by the white precipitation surrounding the colonies. In contrast to their growth on standard TS agar plates devoid of TDC supplementation, the morphology is notably distinct. The formation of this white precipitate around the colonies underscores the enzymatic deconjugation of bile salts to primary bile salts by these strains [56].

### Haemolytic activity

The haemolytic activity of isolated *B. subtilis* SOM8 and wild type strain *B. subtilis* ATCC 6051 was shown in Supplementary Figure S5. Both isolated *B. subtilis* SOM8 and wild-type *B. subtilis* ATCC 6051 exhibited α-haemolytic activity, characterized by partial or green haemolysis linked to the reduction of red cell haemoglobin. This phenomenon is attributed to the production of hydrogen peroxide by the bacterium such as *S. pneumoniae*, causing oxidation of iron in haemoglobin and resulting in the formation of the green oxidized derivative, methaemoglobin [57].

### Cell cytotoxicity using Caco-2 cells (CCK-8 Assay)

**Fig. 8:**
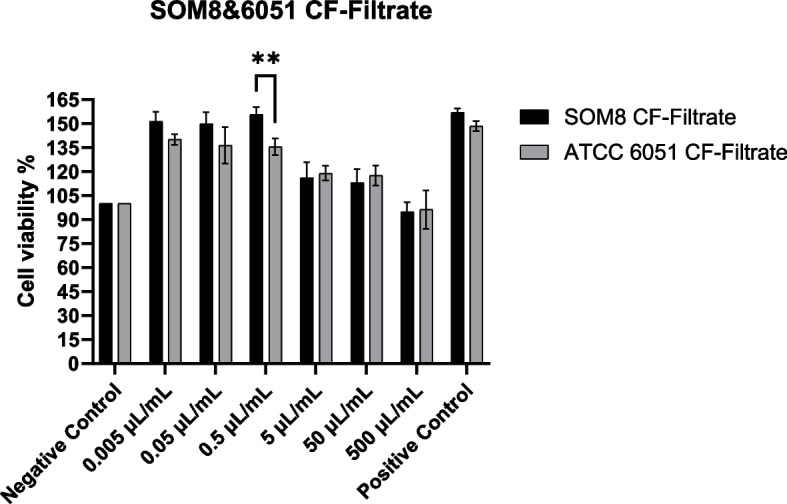
The effects of B. subtilis SOM8 and B. subtilis ATCC 6051 cell-free filtrate on viability of Caco-2 cells

**Fig. 9:**
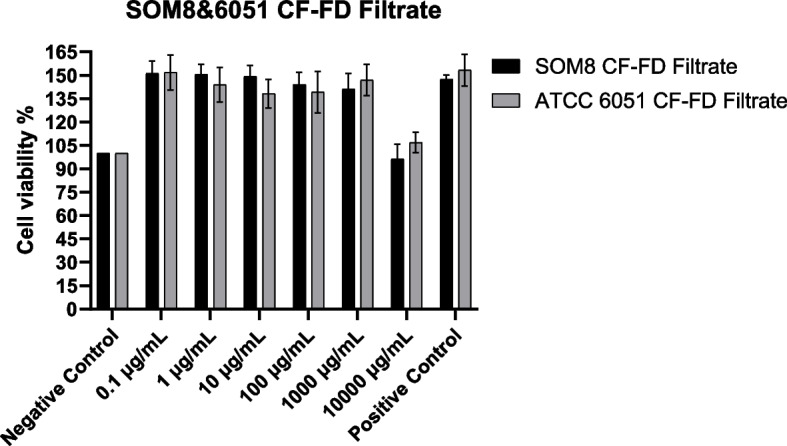
The effects of B. subtilis SOM8 and B. subtilis ATCC 6051 freeze-dried cell-free filtrate on viability of Caco-2 cells

The results depicting cell cytotoxicity resulting from exposure to the cell-free filtrate and lyophilized cell-free filtrate of *B. subtilis* SOM8 and *B. subtilis* ATCC 6051 are illustrated in Figs. 8 and  9, respectively.

Notably, both the cell-free filtrate and freeze-dried filtrate of *B. subtilis* SOM8 and *B. subtilis* ATCC 6051 exhibited low cytotoxicity. At concentrations of 5 µL/mL or 10 mg/mL, an observable trend indicated that the filtrates demonstrated to inhibit the proliferation of Caco-2 cells. This phenomenon might be attributed to the presence of bioactive compounds and hydrogen peroxide produced by both strains of *B. subtilis*. Furthermore, the substitution of the fermented solution for DMEM was identified as a contributing factor to the observed inhibition, as evidenced by a relatively higher inhibitory effect for the cell-free filtrate compared to the freeze-dried filtrate. In summary, considering the low cytotoxicity towards Caco-2 cells, both *B. subtilis* SOM8 and *B. subtilis* ATCC 6051 are viable candidates for probiotic applications, with *B. subtilis* SOM8 exhibiting comparatively better performance.

### Adhesion capacity assay using caco-2 cells

**Fig. 10:**
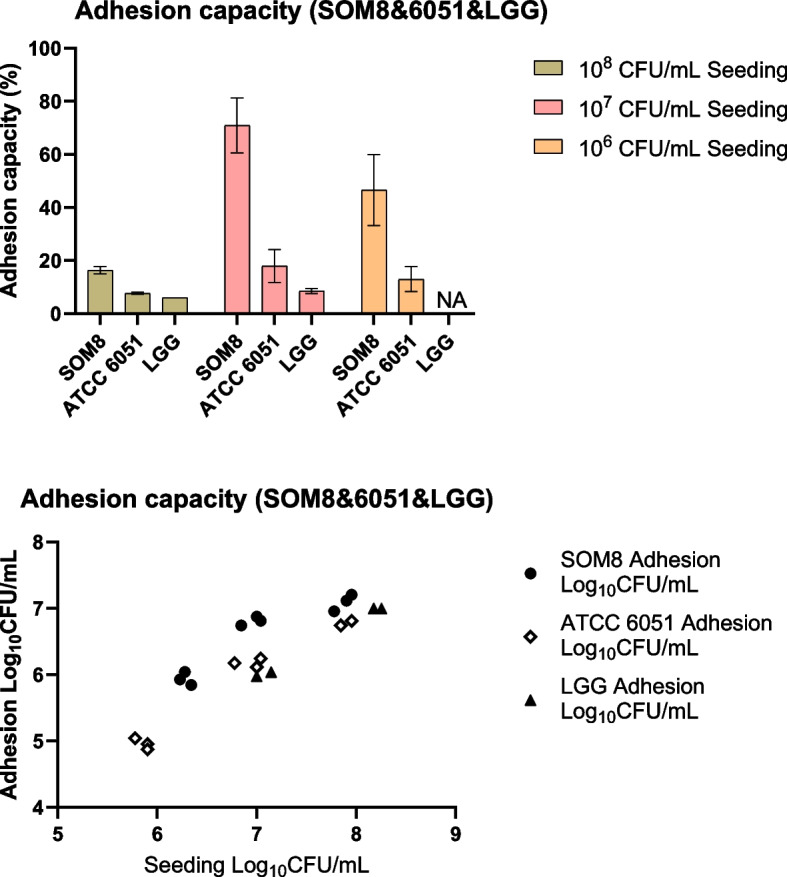
Adhesion capacity of B. subtilis SOM8, B. subtilis ATCC 6051 and LGG to Caco-2 cells under different initial seeding concentrations

The adhesion capabilities of *B. subtilis* SOM8, *B. subtilis* ATCC 6051, and LGG to Caco-2 cells are graphically represented in Fig. 10. Notably, adhesion capacity exhibits no apparent correlation with the initial seeding concentration. Interestingly, all three strains demonstrated optimal adhesion at an initial seeding concentration of 10^7^ CFU/mL, with *B. subtilis* SOM8 owning an approximately 70% adhesion rate, significantly higher than both *B. subtilis* ATCC 6051 of 20% and LGG of 8%. This heightened adhesion exhibited by *B. subtilis* SOM8 is possible to be attributable to its augmented biofilm-producing and intrinsic adhesion properties. Importantly, it is observed that *B. subtilis* SOM8 consistently meets the required criteria for probiotic efficacy, maintaining adhesion values surpassing 10^6^ CFU/mL across varying seeding concentrations. This adherence threshold, as established guidelines [52], indicated *B. subtilis* SOM8's fulfilment of the necessary criteria for optimal probiotic functionality under diverse seeding conditions.

### WGS of B. subtilis SOM8 for Genotypic Characterization

#### Virulence factors identification

The prediction of virulence factors within *B. subtilis* SOM8 was facilitated through the VFDB database. As illustrated in Table 3, the genome of *B. subtilis* SOM8 revealed a total of 10 matches with VF-associated protein. *B. subtilis* SOM8 was observed to lack the *B. cereus* cereulide gene cluster (*cesABCHPT*) and the enzyme genes encoded by pathogenic *Bacillus* species. The virulence factor of *B. subtilis* SOM8 involves toxins, immune evasion, and iron acquisition. Among the identified virulence factors, the iron acquisition, related genes *dhbABCDE* were identified within the genome of *B. subtilis* SOM8. However, these *dhbA-E* genes are a common genetic component in *B. subtilis* subsp. subtilis 168, a strain extensively utilised in industrial applications. Notably, *B. subtilis* SOM8 was also predicted to encode for *capABCD* genes, implicated in polyglutamate synthesis and transport. However, the *capE* gene, present in *B. anthracis* and *B. cereus biovar anthracis*, is absent. The final identified virulence factor is toxins. The *B. subtilis* SOM8 was predicted to encode a haemolysin, putative membrane hydrolase (*hlyIII*), based on genetic information. Notably, comparable haemolytic activity has been detected across several *Bacillus* strains, including those utilised as commercial probiotics [58]. Furthermore, the likelihood of an orally administered probiotic translocating through the intestinal barrier into the bloodstream remains limited and has been reported only at minimal frequencies in hospitalized patients [59].

**Table 3:** Summary of isolated *B. subtilis* SOM8 genome for virulence factor prediction using VFDB database

Virulence factors	Category	Related genes
Toxin	Haemolysin III	*hlyIII*
Immune evasion	Polyglutamic acid capsule	*capA*
		*capB*
		*capC*
		*capD*
Iron acquisition	Bacillibactin	*dhbA*
		*dhbB*
		*dhbC*
		*dhbD*
		*dhbE*

In the comparative analysis of VFs between isolated *B. subtilis* SOM8 and *B. subtilis* ATCC 6051 a striking similarity in VFs was observed. However, it was found that *B. subtilis* SOM8 lacks the *bpsC* gene responsible for *B. cereus* EPS production that exists in *B. subtilis* ATCC 6051, a specific immune evasion VF present in *B. cereus*. This absence of the *bpsC* gene in *B. subtilis* SOM8 highlights its enhanced safety profile compared to *B. subtilis* ATCC 6051.

### Antibiotic resistance genes identification

The evaluation of antibiotic resistance genes within *B. subtilis* SOM8 was undertaken utilizing the CARD. Out of a total of 274 hits, 10 hits exhibited a minimum identity of 95% and were subsequently categorized as strict matches, as shown in Supplementary Table S4. Notably, the remaining hits displaying identity levels below 80% were not taken into consideration. For instance, with an identity of 98.59% to the *aadK* gene, the isolated *B. subtilis* SOM8 is predicted to exhibit resistance against streptomycin. Additionally, its resistance to macrolides spiramycin and telithromycin can be attributed to a 98.35% identity with the gene *mphK*, which encodes a macrolide phosphotransferase. In summary, *B. subtilis* SOM8 was predicted to harbour 10 antibiotic resistance genes, conferring potential resistance against a diverse spectrum of antibiotics. These encompass peptides, fluoroquinolones, aminoglycosides, tetracyclines, phenicols, lincosamides, nucleosides, macrolides, streptogramins antibiotics as well as disinfecting agents and antiseptics. Nevertheless, it is imperative to acknowledge that gene prediction does not necessarily imply gene expression. To address this critical aspect, MIC assessments against a spectrum of medically significant antibiotics were conducted.

### Plasmid and MGEs Identification

Drawing from the outcomes obtained through the PlasmidFinder 2.1 [40] and MobileElementFinder [41], it is evident that *B. subtilis* SOM8 lacks plasmid genes and any MGEs. The inference can be drawn that this strain may not possess the capability to transfer potential antibiotic resistance genes to other bacterial entities. It is necessary to acknowledge, however, that these conclusions stem from BLAST-based assessments and genetic data. For a more comprehensive understanding, the need for in vivo investigations or subsequent clinical trials is still necessary, especially in anticipation of the eventual integration of this strain into both industrial and medical applications.

### MIC Evaluation of B. subtilis SOM8

The sensitivity of *B. subtilis* SOM8 to eight medically prescribed antibiotics was tested following CMSI and EFSA MIC standard, the results were shown in Table 4. The investigation revealed that isolated *B. subtilis* SOM8 displayed susceptibility to seven out of eight common antibiotics, including one glycopeptide, two aminoglycosides, two macrolides, one tetracycline, and one phenicol antibiotic, in accordance with EFSA standards. The MIC of *B. subtilis* SOM8 to streptomycin was approximately 128 μg/mL, notably exceeding the EFSA threshold. However, it is essential to notify that resistance to streptomycin is generally regarded as an intrinsic property of *Bacillus* species that contain the putative *aadK* genes. Moreover, there is no supporting evidence indicating the potential horizontal transfer of such genes to other bacterial strains [60].

**Table 4:** MIC results of isolated *B. subtilis* SOM8 against eight common antibiotics

Antibiotics	Function	Type	MIC (${\varvec{\upmu}}$ g/mL)	EFSA Threshold (${\varvec{\upmu}}$ g/mL)
Vancomycin	Cell wall synthesis	Glycopeptide	0.25	4
Gentamicin	Protein synthesis (30S)	Aminoglycosides	1	4
Kanamycin	Protein synthesis (30S)	Aminoglycosides	8	8
Streptomycin	Protein synthesis (50S)	Aminoglycosides	128	8
Erythromycin	Protein synthesis (50S)	Macrolides	0.25	4
Clindamycin	Protein synthesis (50S)	Macrolides	2	4
Tetracycline	Protein synthesis (30S)	Tetracycline	0.25	8
Chloramphenicol	Protein synthesis (50S)	Phenicol	4	8

### Secondary Metabolites (antiSMASH, BAGEL4) Prediction

The assessment of secondary metabolites, including bacteriocins, synthesized by the isolated *B. subtilis* SOM8 was conducted using the antiSMASH and BAGEL4 databases, as detailed in Table 5. *B. subtilis* SOM8 was predicted to yield six distinct secondary metabolites, encompassing both Ribosomally Synthesized and Post-Translationally Modified Peptides (RiPPs) and Non-Ribosomal Peptide Synthases (NRPS). These include fengycin, bacillaene, subtilosin, bacilysin, bacillibactin, and lichendicin.

**Table 5:** Summary of predicted secondary metabolites produced by isolated *B. subtilis* SOM8

Type	Most similar cluster	% Similarity
NRPS	Fengycin	93
Polyketide + NRPS	Bacillaene	100
RiPPs: Thiopeptide	Subtilosin A	100
Other	Bacilysin	100
NRPS	Bacillibactin	100
RiPPs: Lanthipeptide	Lichendicin A1	96

In contrast to primary metabolites, these secondary metabolites represent non-essential, small organic molecules that can potentially confer evolutionary advantages over time, such as enhancing survival in competition with other organisms. *B. subtilis* SOM8 is predicted to engage in the synthesis of diverse bioactive molecules, notably encompassing various antibiotics with considerable potential for applications. These findings underscore the SOM’s capacity to produce an array of compounds with potential therapeutic applications.

### Comparison with Wild Type Strain B. subtilis ATCC 6051

The growth patterns of *B. subtilis* SOM8 and the wild-type strain *B. subtilis* ATCC 6051 were compared under both aerobic and anaerobic conditions, as depicted in Supplementary Figure S6. Additionally, their respective antipathogenic activities against the specified human enteropathogens were tested under both aerobic and anaerobic conditions, as presented in Supplementary Figure S7. The results underscored *B. subtilis* SOM8's superior growth performance under both aerobic and anaerobic conditions. Notably, the *B. subtilis* SOM8 colonies exhibited enhanced dimensions and were surrounded by a more substantial excretion of biofilm, a matrix of extracellular substances known to create a favourable microenvironment for bacterial proliferation, particularly in challenging conditions.

The outcomes from Supplementary Figure S7 emphasize that both isolated *B. subtilis* SOM8 and *B. subtilis* ATCC 6051 own a wide spectrum of antipathogenic activities against several pathogens. However, it is noteworthy that *B. subtilis* SOM8 outperforms *B. subtilis* ATCC 6051 under anaerobic conditions. Specifically, when cultivated under anaerobic conditions (represented by Number 5 and 6), *B. subtilis* ATCC 6051 exhibits a loss of antipathogenic efficacy against *V. parahaemolyticus* and *S. aureus*, whereas *B. subtilis* SOM8 sustains its robust inhibition of pathogen growth, as evidenced by the inhibition zones.

## Discussion

Over a long period, numerous strains within the *Bacillaceae* family, such as *B. subtilis*, *B. licheniformis*, and *B. coagulans*, have found application as probiotics in dietary supplements for both human consumption and animal feed [61]. Nevertheless, it is vital to ensure safety when considering *Bacillaceae* species as probiotics. This is especially so given that certain members, including *B. anthracis* and *B. cereus*, are pathogenic to both humans and animals [62]. Here, we present evidence that supports the candidacy of isolated *B. subtilis* SOM8, sourced from food processing waste—SOM, as a potential probiotic strain. *B. subtilis* SOM8 has great potential for inhibiting human enteropathogens, it is also equipped with robust stress tolerance, beneficial host-associated attributes, and an evidently safe preclinical profile.

The observed stress tolerance of *B. subtilis* SOM8 to acidic conditions, bile salts, and its heat stability can be ascribed to its inherent capacity for biofilm production. The self-produced biofilm serves as a shield, not only contributes to its stress tolerance but also imparts mucoadhesive properties, thus enhancing its utility in biomedical and nutraceutical applications [63]. Moreover, it is assumed that its performance in tolerating harsh conditions is superior to what was observed in this test. During the stress tests, certain actions, such as pipetting and vortexing, may have disrupted the original biofilm structure produced by *B. subtilis* SOM8, potentially reducing its effectiveness in withstanding challenging environments. Nonetheless, *B. subtilis* SOM8 still exhibited commendable performances.

BSH activity of *B. subtilis* SOM8 accounts for its cholesterol lowering potential. BSH enzymes can catalyse a reaction involving the cleavage of the peptide linkage within bile acids, the resulting unconjugated bile acids exhibit decreased solubility and tend to precipitate under acidic conditions. As a result, larger quantities of free bile acids will be excreted in faeces. Such deconjugation could increase the demand for cholesterol as a substrate for the de novo synthesis of bile acids, compensating for the loss of bile acids excreted in faeces. This elevated demand for cholesterol may result in a reduction in circulating cholesterol levels. In addition, the deconjugation of bile salts may decrease in the solubility of cholesterol, thereby impeding its absorption across the intestinal lumen. As a consequence, the overall absorption of cholesterol from the gut is diminished [56].

Antioxidants have gained significant interest due to their numerous benefits, including anti-aging and anti-inflammatory properties. In the area of food technology, antioxidants are incorporated into a wide range of food products to enhance their nutritional value. The antioxidant activities of *B. subtilis* SOM8 makes it promising for its use in the prevention and treatment of diseases in the area of pharmacology, cosmetics, and medicine area [64].

With regards to the virulence factors associated with *B. subtilis* SOM8, it is notable that the products encoded by these genes exhibited the absence of intrinsic toxicity. For instance, the catecholate siderophore Bacillibactin, a secondary metabolite encoded by the *dhb* operon, is responsible for chelating and facilitating the utilization of ferric ions. The iron acquisition potential of Bacillibactin has garnered interest in applications beyond pathogenesis, including its role in addressing iron accumulation in the substantia nigra of the brain, thereby holding promise for the treatment of conditions such as Parkinson's disease [65]. *B. subtilis* SOM8 genome also encodes the *capABCD* genes, responsible for polyglutamate synthesis and transport. Notably, polyglutamate has been implicated in enhancing the pathogenicity of *B. anthracis* by evading the host's innate immune response. However, it is important to emphasize that polyglutamate production is a characteristic shared by numerous commensal *Bacillus* strains, including commercially utilised strains such as *B. licheniformis* and *B. subtilis* subsp. subtilis 168. Furthermore, the presence of polyglutamate is a common occurrence in various foods subjected to fermentation processes involving *Bacillus* species [66], indicating its intrinsic nature. Furthermore, *B. subtilis* SOM8 lacks the *capE* gene found in pathogenic species, further substantiating its safer profile.

The cytotoxicity of *B. subtilis* SOM8 were investigated using Caco-2 cells models, the findings revealed that both the cell-free filtrate and freeze-dried cell-free filtrate exhibited low cytotoxicity towards Caco-2 cells. Nevertheless, upon the increased concentration, an inhibitory trend on cell proliferation emerged, attributed to the presence of bacteriocin and other substances, such as hydrogen peroxide, exerting cytotoxic effects on the cells. It is noteworthy that previous study has also proved the cytotoxic impact of commercial LAB, including LGG, *L. casei* M3, and *L. plantarum* YYC-3, along with their metabolite secretions, on colon cancer cells such as Caco-2 and HT-29 [67]. This observation suggests an inherent anti-cancer potential in a distinct context, highlighting the multi-faceted nature of bacterial interactions with colon cancer cells.

Isolated *B. subtilis* SOM8 demonstrated α-haemolytic activity, raising potential safety concerns for its application in human consumption or animal nutrition. Nevertheless, considering the precedent application of various *Bacillus* strains [68, 69], particularly *B. subtilis* ATCC 6051 [70], it is observed that only strains exhibiting β-haemolytic activity are discouraged for further application. Moreover, even in many *Lactobacilli* probiotic products, such as kefir isolates [34, 71], the presence of toxin protein *hlyIII* is common and has not been considered a significant concern. Additionally, cases of bacteremia demonstrating the transmission of the probiotic from the product to the blood are infrequent to be identified [59], suggesting a low likelihood of an oral probiotic translocating through the intestinal barrier into the bloodstream [10]. In addition, we have proved *B. subtilis* SOM8 low cytotoxicity using Caco-2 cell line models.

AMR mechanisms have undergone changes through bacterial evolution. Certain mechanisms have primarily emerged to bacteria against natural antimicrobial agents, whereas others have evolved for distinct cellular functions. These mechanisms are commonly denoted as intrinsic mechanisms. It is noteworthy that intrinsic resistance mechanisms usually do not spread horizontally among bacteria; instead, they tend to proliferate clonally. Therefore, when a bacterial species exhibits inherent resistance to an antimicrobial, denoted as 'intrinsic resistance,' a characteristic prevalent among all strains of that species, is generally not considered as a safety concern. In contrast, when a strain of a species typically susceptible to a specific antimicrobial demonstrates resistance to that drug, it is categorized as 'acquired resistance.' Such acquired resistance warrants further in-depth investigation [45]. The prevalence of streptomycin resistance is a phenomenon that spans across a wide spectrum of *Bacillus* species, and it is highly probable that this resistance is an inherent characteristic rather than acquired resistance from mobile genetic elements [10, 15]. As such, the observed resistance of *B. subtilis* SOM8 to streptomycin is not considered as a serious safety concern.

Furthermore, *B. subtilis* SOM8 is predicted to engage in the synthesis of diverse bioactive molecules, notably encompassing various antibiotics with considerable potential for applications. These findings underscore its capacity to produce an array of promising compounds with potential therapeutic applications. For instance, Fengycin has exhibited antimicrobial properties in preclinical studies and has been suggested as bioactive in clinical observational trials to combat pathogens like *S. aureus* [72]. Bacilysin, a dipeptide antibiotic, has demonstrated efficacy in inhibiting Gram-negative foodborne pathogens [73], while bacillaene, a polyene antibiotic, displays broad-spectrum antimicrobial activity against pathogens including *S. aureus and E. coli* [74]. Moreover, bacillaene has the additional capacity to promote biofilm formation [75]. Subtilosin A, another secondary metabolite predicted to be produced by isolated *B. subtilis* SOM8, is characterized by its remarkable resistance to enzymatic proteolysis and its stability under moderate heat and acid conditions. It has demonstrated efficacy against various Gram-positive bacteria, including *Listeria* [76, 77]. Lastly, lichendicin, categorized as a lantibiotics, showcases antimicrobial activities against a spectrum of strains including *Listeria* monocytogenes, *S. aureus*, and vancomycin-resistant *Enterococcus* [78].

## Conclusion

Given the results of conducted screening assays, including both phenotypic and genotypic assessments, the isolated *B. subtilis* SOM8 strain exhibits a safe preclinical profile. These findings support the potential utility of *B. subtilis* SOM8 as a viable candidate for applications as probiotics for human consumption, including dietary supplements, nutraceuticals, and medical purposes.

### Supplementary Information

**Supplementary Material 1.**

## Data Availability

The raw fastq data of *B. subtilis* SOM8 can be available at NCBI Sequence Read Archive (SRA) website database using the given NCBI/Genbank accession number PRJNA1009692. Link: (https://www.ncbi.nlm.nih.gov/sra/PRJNA1009692). The assembled fasta data of *B. subtilis* SOM8 can be available at NCBI Nucleotide database using the given NCBI/Genbank accession number JAVICJ000000000. Link: (https://www.ncbi.nlm.nih.gov/nuccore/JAVICJ000000000).

## Abbreviations

SOM: Sesame oil meal
SGF: Simulated gastric fluids
SIF: Simulated intestinal fluids
BSH: Bile salt hydrolase
MIC: Minimum inhibitory concentration
FAO: Food and Agriculture Organization
WHO: World Health Organization
CAGR: Compound Annual Growth Rate
LAB: Lactic acid bacteria
GIT: Gastrointestinal tracts
GRAS: generally recognized as safe
AMR: Antimicrobial resistance
ATCC: American Type Culture Collection
SCELSE: Singapore Centre for Environmental Life Science Engineering
MRS: De Man Rogosa and Sharpe
TS: Tryptic Soy
NB: Nutrient broth
DPPH: 2,2-diphenyl-1-picrylhydrazyl
DMEM: Dulbecco’s Modified Eagle Medium
FBS: fetal bovine serum
EDTA: ethylenediaminetetraacetic acid
PBS: phosphate-buffered saline
NCBI: National Centre for Biotechnology Information
WGS: Whole Genome Sequencing
DFAST: DDBJ Fast Annotation and Submission Tool
VFDB: Virulence factor database
CARD: Comprehensive Antibiotic Resistance Database
MGEs: Mobile Genetic Elements
TYGS: Type Strain Genome Server
CLSI: Clinical and Laboratory Science Institute
EFSA: European Food Safety Authority
CFU: colony forming units; AA%: antioxidant activity percentage
TDC: taurodeoxycholate hydrate
CCK-8: Cell Counting Kit 8
NEAA: Non-Essential Amino Acids
SD: standard deviation
LGG: L. rhamnosus GG
EPS: exopolysaccharide
RiPPs: Ribosomally Synthesized and Post-Translationally Modified Peptides
NRPS: Non-Ribosomal Peptide Synthases
