# Supplementary material for: Bacillus subtilis SOM8 isolated from sesame oil meal for potential probiotic application in inhibiting human enteropathogens
Source: BMC Microbiol. 2024 Mar 28;24:104. doi: 10.1186/s12866-024-03263-y (PMC11312844; doi:10.1186/s12866-024-03263-y)
Supplement: Supplementary file 1 — Supplementary Material 1. [file 12866_2024_3263_MOESM1_ESM.docx]

Supplementary Materials for

***Bacillus subtilis SOM8 Isolated from Sesame Oil Meal for Potential Probiotic Application in Inhibiting Human Enteropathogens***

**Zhongtian Zhao ^1^, Wenrui Li ^1^, Tran The Thien ^1^, Say Chye Joachim Loo ^1, 2, 3, #^**

^#^ Correspondence to: joachimloo@ntu.edu.sg

**This PDF file includes:**

Supplementary Figure S1 to S7; Table S1 to S4


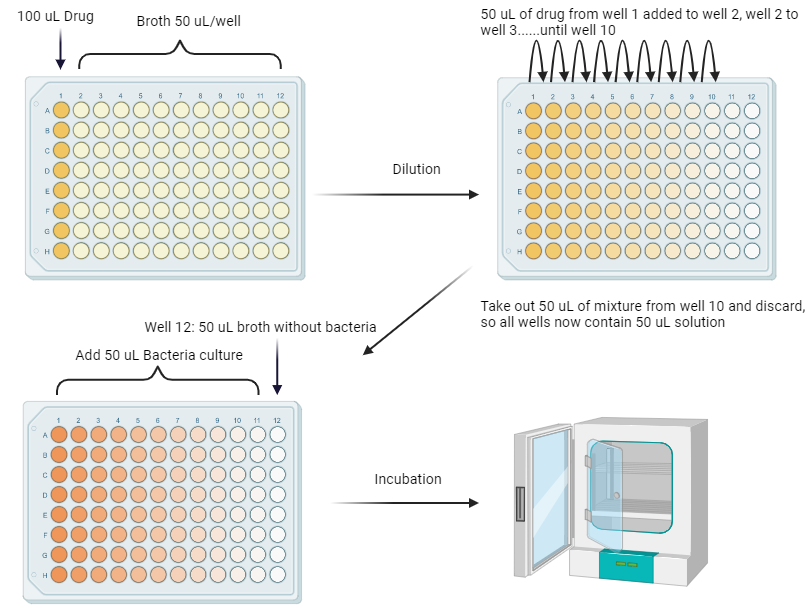


***Figure S1.*** The protocol of CLSI M07 MIC evaluation.


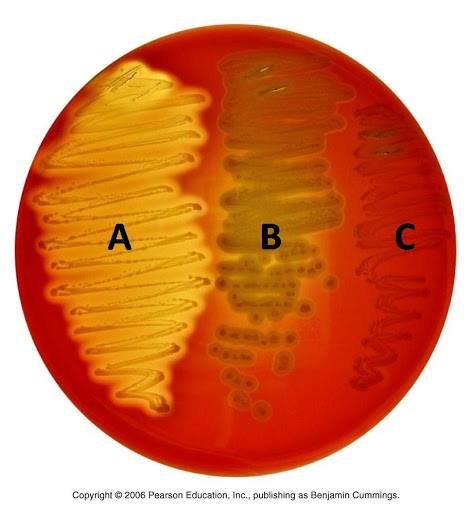


***Figure S2.*** Three types of hemolytic activity (β, α, γ respectively from A to C).

1. *B. cereus* ATCC11778

**
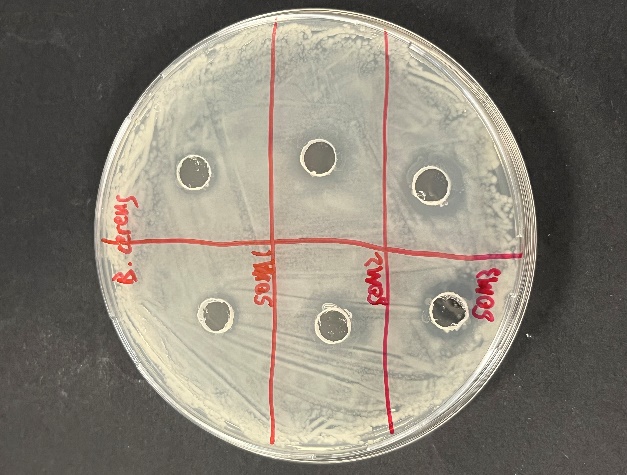

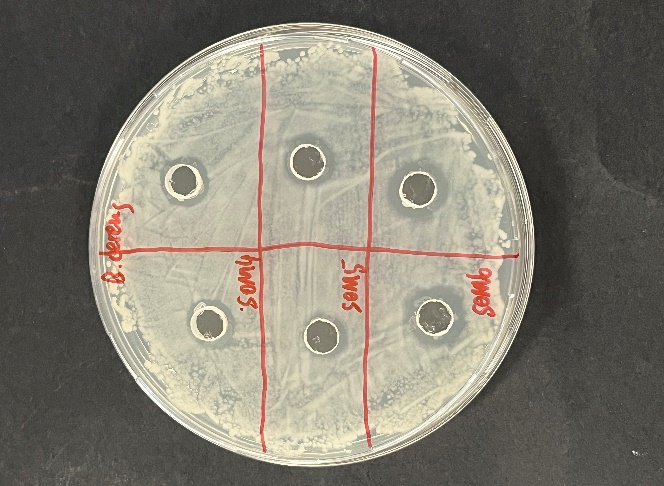

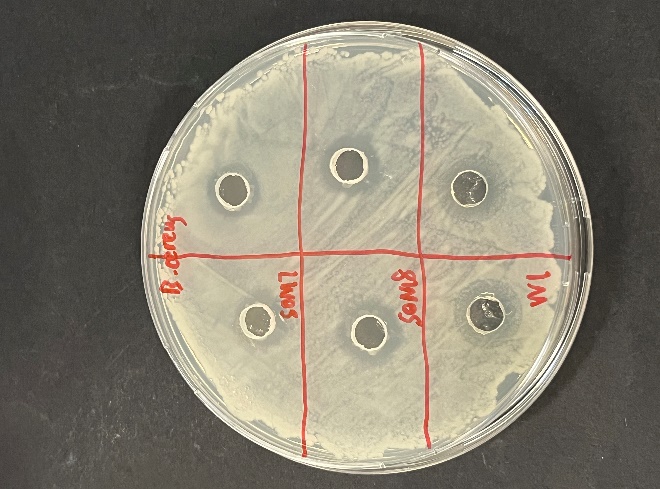
**

1. *E. Coli* O157:H7 ATCC43888


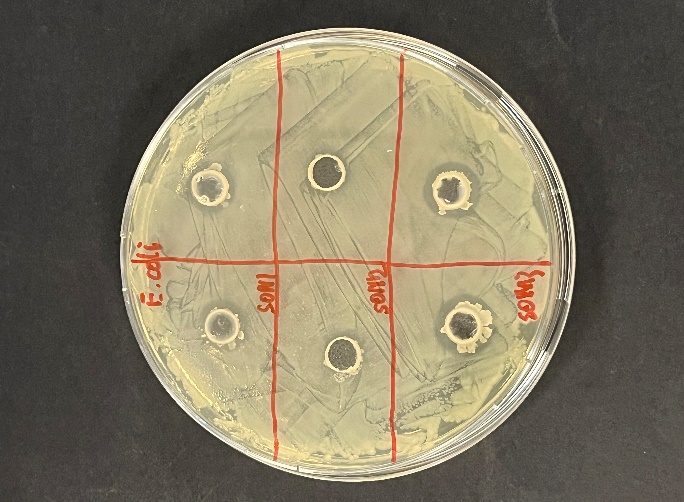

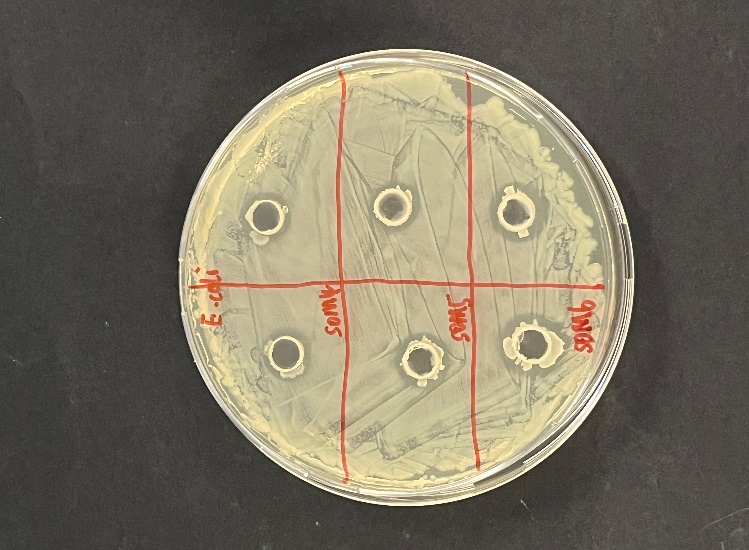

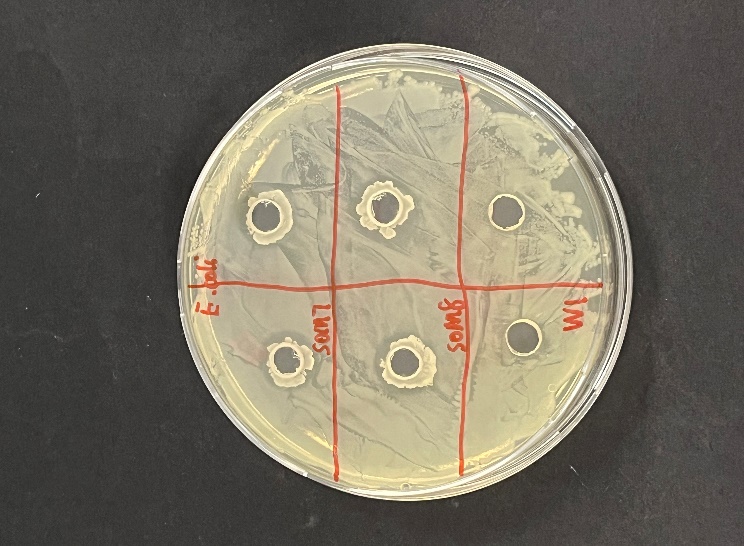


1. *S. aureus* USA300


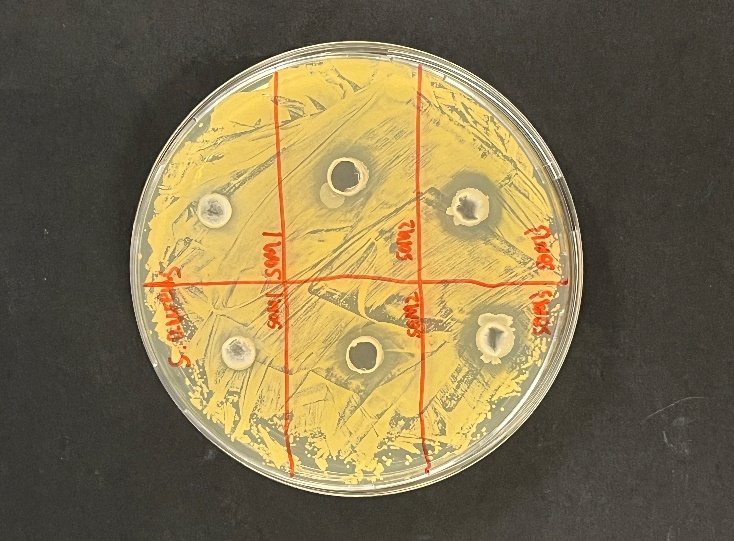

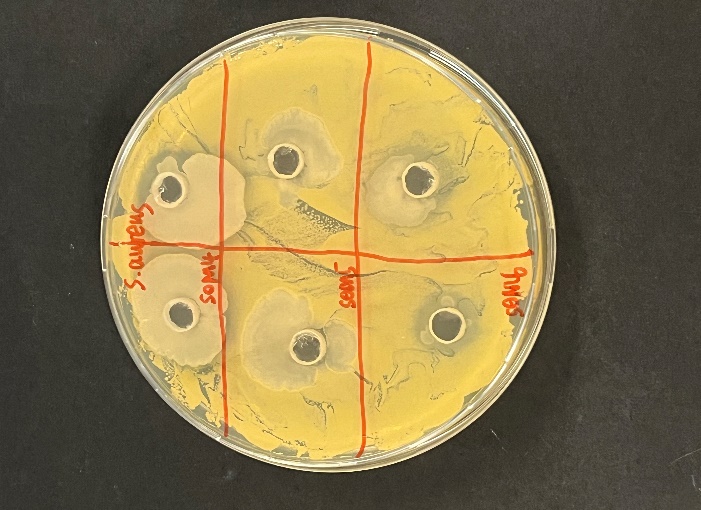

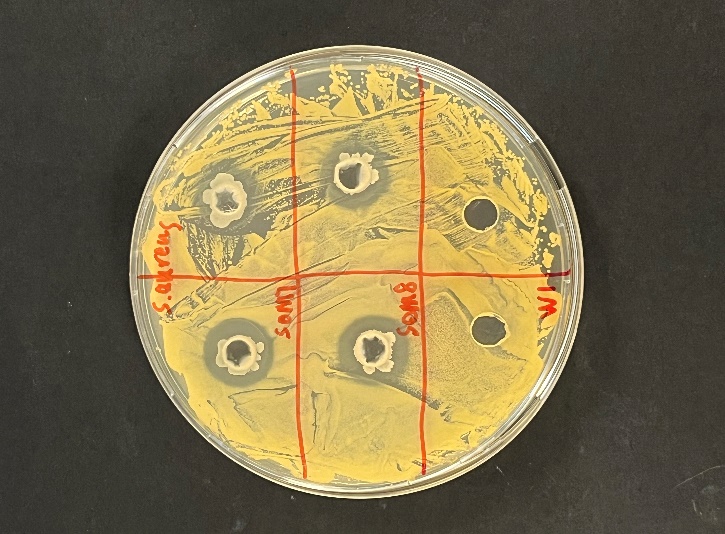


1. *S. enterica* subsp. enterica ATCC BAA-190


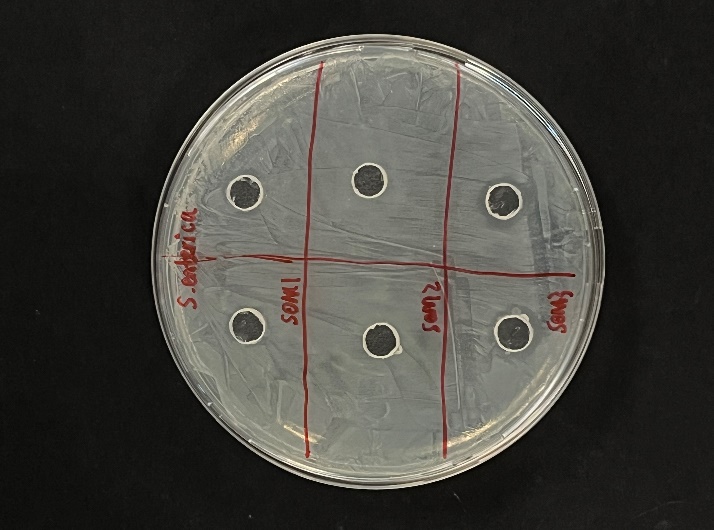

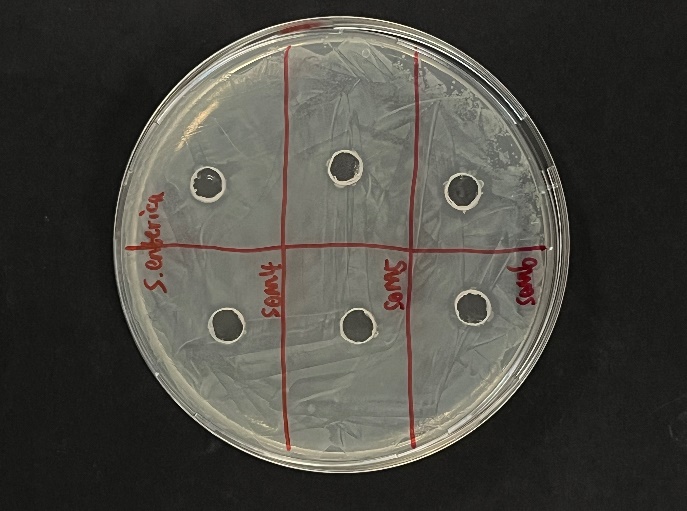

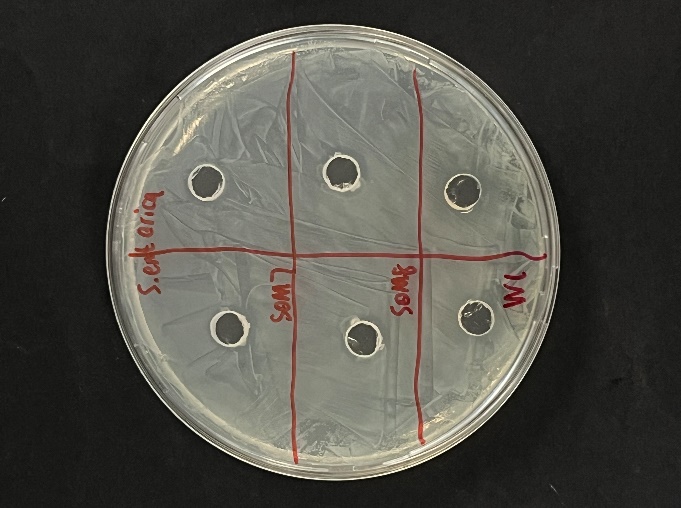


1. *V. parahaemolyticus* ATCC 17802

**
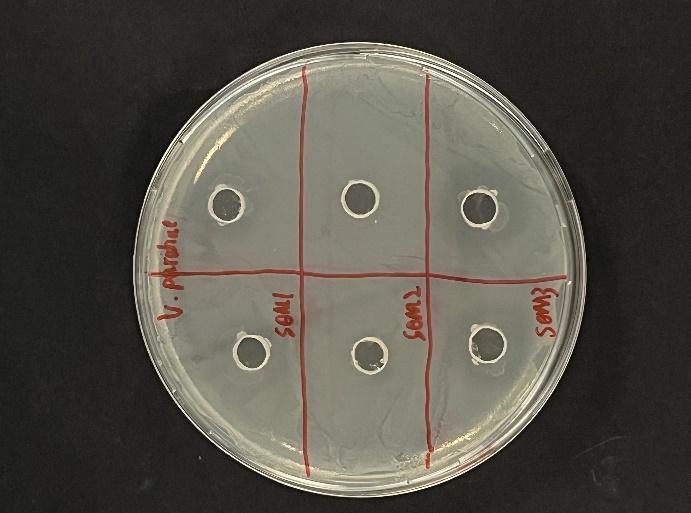

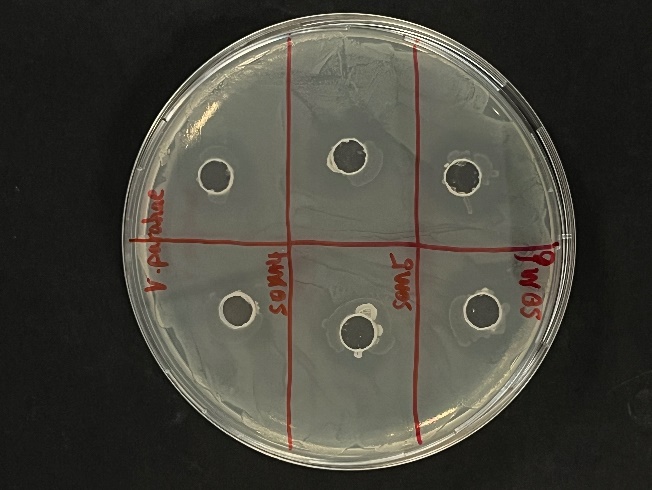

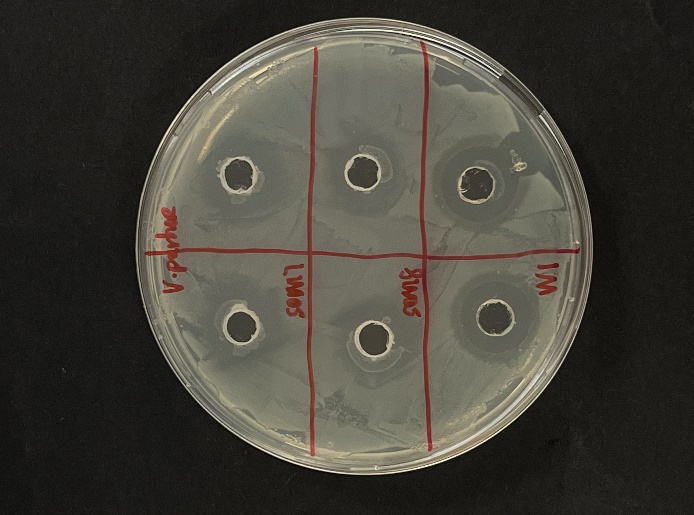
**

***Figure S3.*** Antipathogenic activities of *B. subtilis* SOM1-8 and *W. paramesenteroides* (W1) to inhibit five enteropathogens through agar well diffusion assay.

**Table S1.** Isolated strains’ inhibition to human enteropathogens.

| **Species** | **Strain** | **Diameter of Inhibition Zone (mm)** | | | | |
| --- | --- | --- | --- | --- | --- | --- |
|  |  | ***S. aureus*** | ***E. coli* O157:H7** | ***B. cereus*** | ***S. enterica*** | ***V. parahaemolyticus*** |
| ***B. subtilis*** | **1** | 1.2 | 2.2 | 1.2 | 1.0 | 0.8 |
|  |  | - | 2.5 | 1.5 | 1.2 | - |
|  | **2** | 3.6 | 1.5 | 3.8 | 0.5 | - |
|  |  | 4.5 | 1.5 | 3.5 | - | 0.5 |
|  | **3** | 6.2 | 4.5 | 4.8 | 2.0 | 3.5 |
|  |  | 6.5 | 4.2 | 5.0 | 2.0 | 3.8 |
|  | **4** | 4.2 | 3.0 | 2.5 | 1.0 | 2.5 |
|  |  | 3.2 | 3.5 | 3.0 | 1.5 | 3.5 |
|  | **5** | 1.0 | 3.0 | 2.0 | 0.5 | 5.0 |
|  |  | 0.5 | 2.5 | 2.5 | 1.0 | 4.2 |
|  | **6** | 3.5 | 4.5 | 4.8 | 2.2 | 3.0 |
|  |  | 3.0 | 4.0 | 4.5 | 2.5 | 3.5 |
|  | **7** | 4.5 | 2.5 | 4.0 | 1.5 | 6.5 |
|  |  | 3.8 | 2.5 | 4.5 | 1.5 | 6.0 |
|  | **8** | 5.0 | 4.0 | 5.0 | 2.5 | 6.5 |
|  |  | 4.5 | 3.8 | 4.8 | 2.5 | 6.5 |
| ***Weissella paramesenteroides*** | **1** | - | 1.0 | 4.8 | 1.0 | 8.0 |
|  |  | - | 1.0 | 3.8 | 1.8 | 7.5 |

**Table S2.** Strains in TYGS dataset


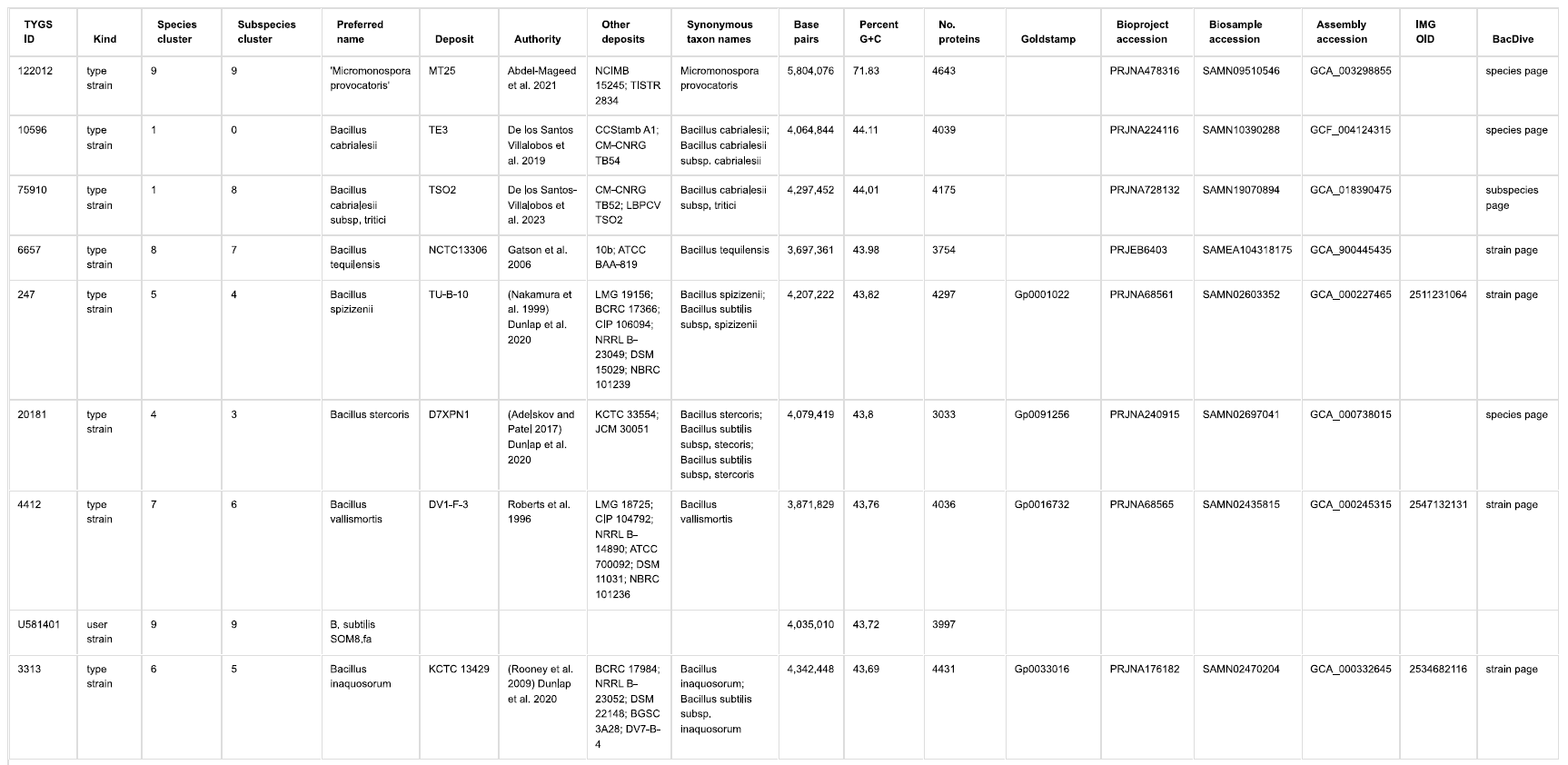


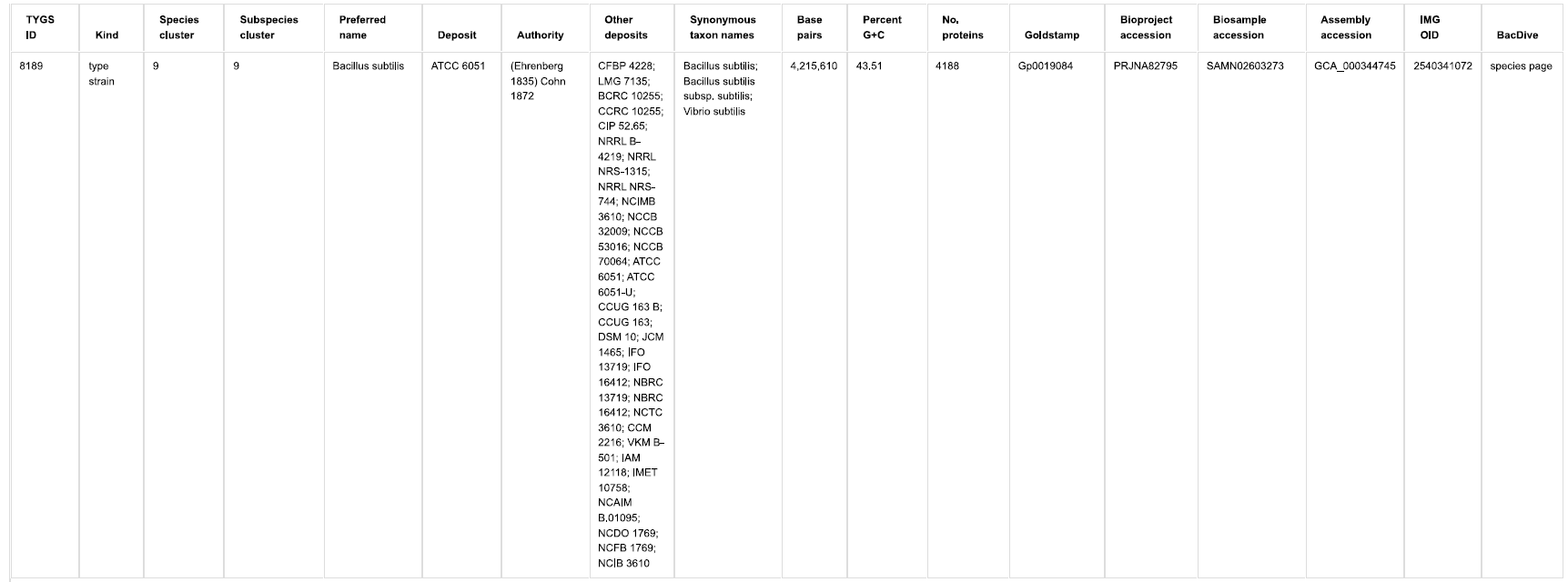


**Table S3.** Pairwise comparisons of user genomes B. subtilis SOM8 vs. type strain genomes


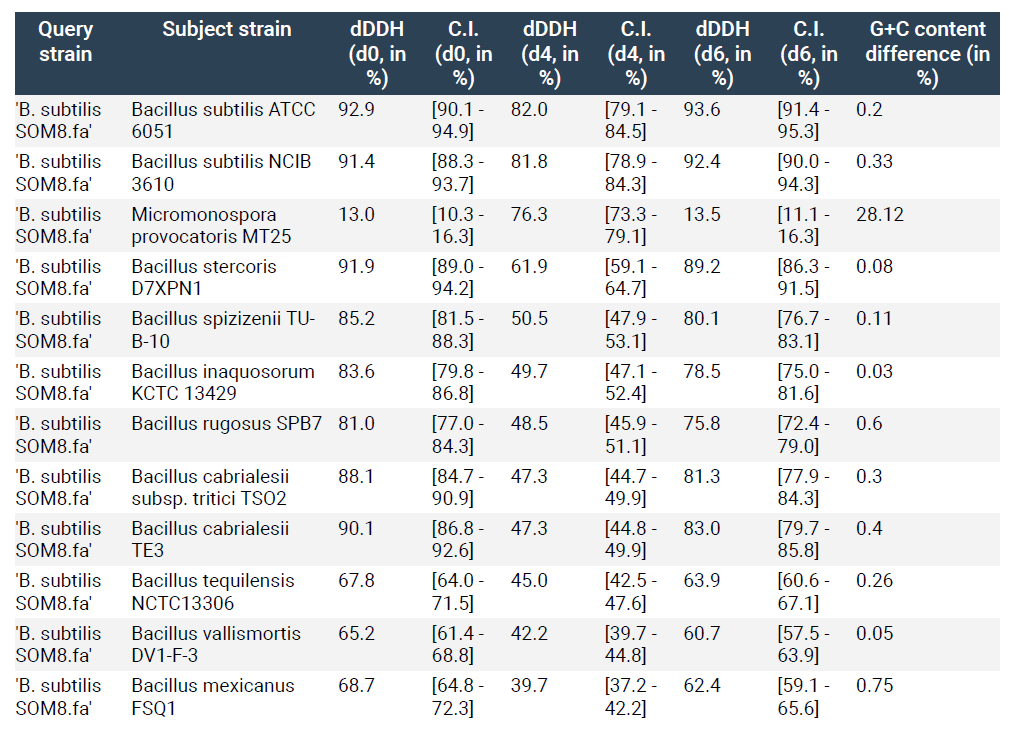


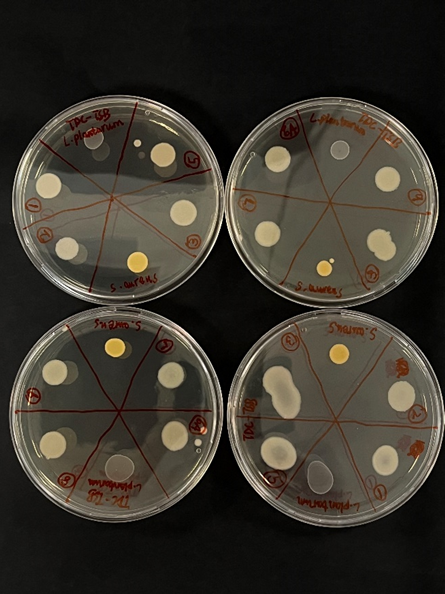

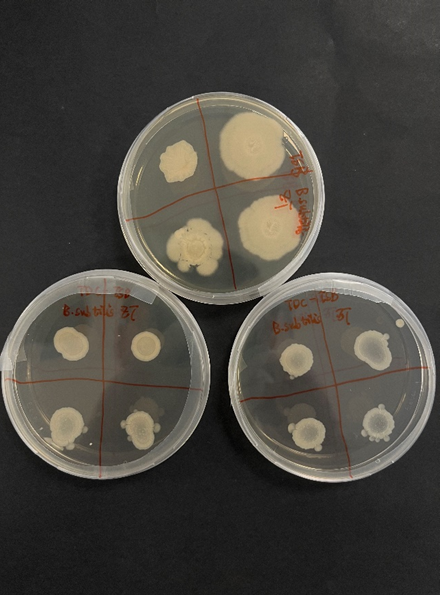


**Figure S4.** BSH activity of isolated B. subtilis SOM (1-8). On the left, duplicates of B. subtilis SOM8 (1-8) are presented alongside S. aureus and L. plantarum, serving as the negative and positive controls, respectively. On the right, B. subtilis SOM8 growth on TDC-TS agar plates is displayed in duplicates, compared with growth on normal TS agar plates without TDC.


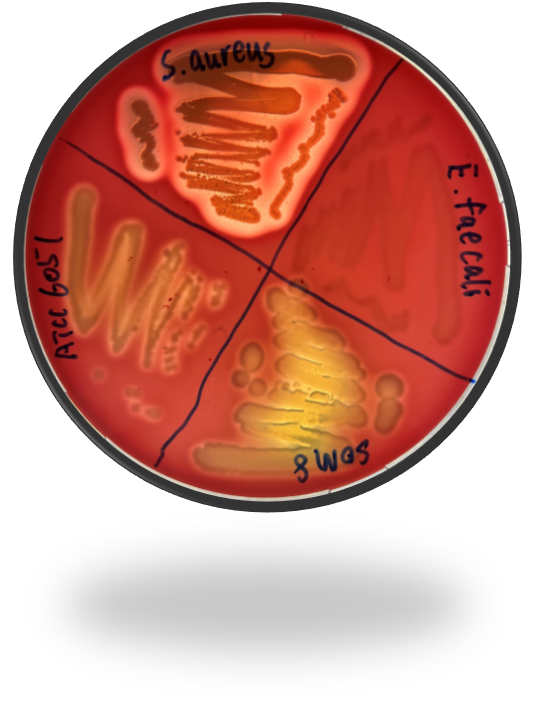

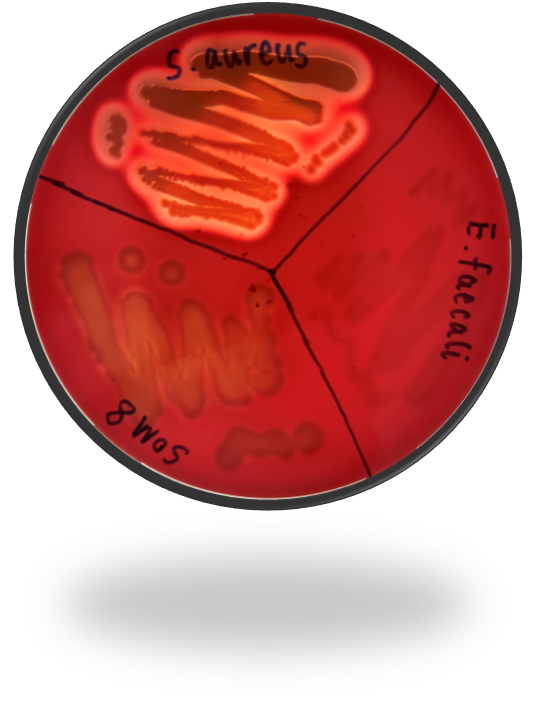


**Figure S5.** Hemolytic activity of B. subtilis SOM8 and B. subtilis ATCC 6051.

**Table S4.** Summary of B. subtilis SOM8 genome for antibiotic resistance prediction.

| **Gene** | **Gene Family** | **Drug Class** | **% Identity** |
| --- | --- | --- | --- |
| B, subtilis mprF | defensin resistant mprF | peptide antibiotic | 99.88 |
| blt | major facilitator superfamily (MFS) antibiotic efflux pump | fluoroquinolone antibiotic, disinfecting agents and antiseptics | 99.75 |
| *ykkC* | small multidrug resistance (SMR) antibiotic efflux pump | aminoglycoside antibiotic, tetracycline antibiotic, phenicol antibiotic | 99.11 |
| *ykkD* | small multidrug resistance (SMR) antibiotic efflux pump | aminoglycoside antibiotic, tetracycline antibiotic, phenicol antibiotic | 99.05 |
| *lmrB* | ATP-binding cassette (ABC) antibiotic efflux pump | lincosamide antibiotic | 98.74 |
| *bmr* | major facilitator superfamily (MFS) antibiotic efflux pump | fluoroquinolone antibiotic, nucleoside antibiotic, phenicol antibiotic, disinfecting agents and antiseptics | 98.71 |
| *aadK* | ANT(6) | aminoglycoside antibiotic | 98.59 |
| *mphK* | macrolide phosphotransferase (MPH) | macrolide antibiotic | 98.35 |
| *vmlR* | Miscellaneous ABC-F subfamily ATP-binding cassette ribosomal protection proteins | lincosamide antibiotic, streptogramin antibiotic, streptogramin B antibiotic | 98.18 |
| *tmrB* | tunicamycin resistance protein | nucleoside antibiotic | 96.95 |

a)
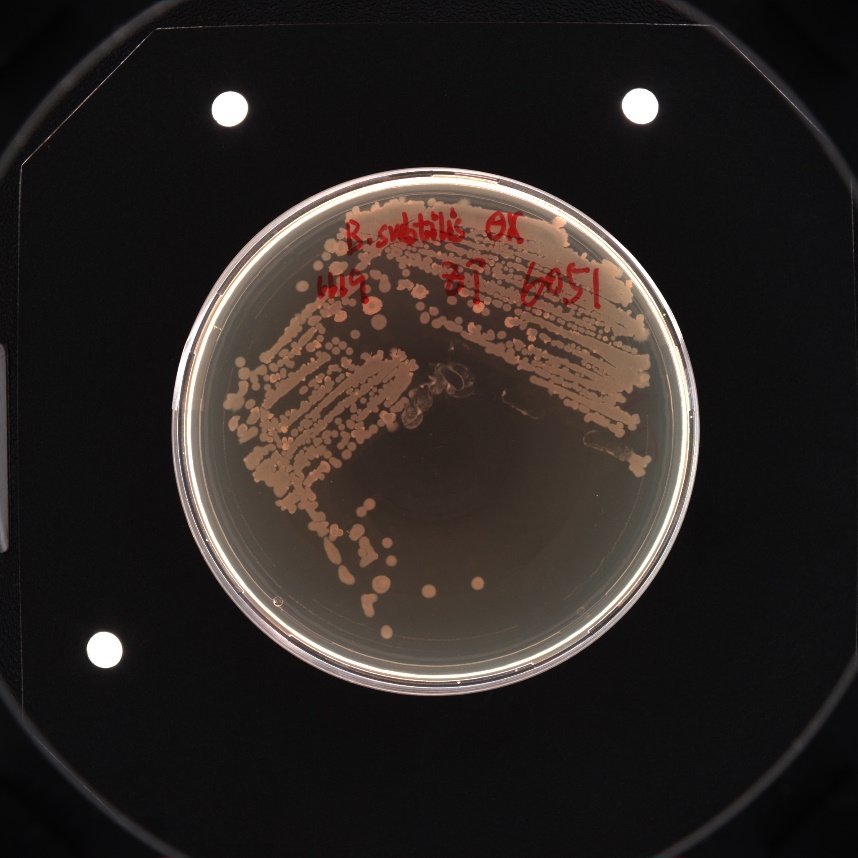
 (b)
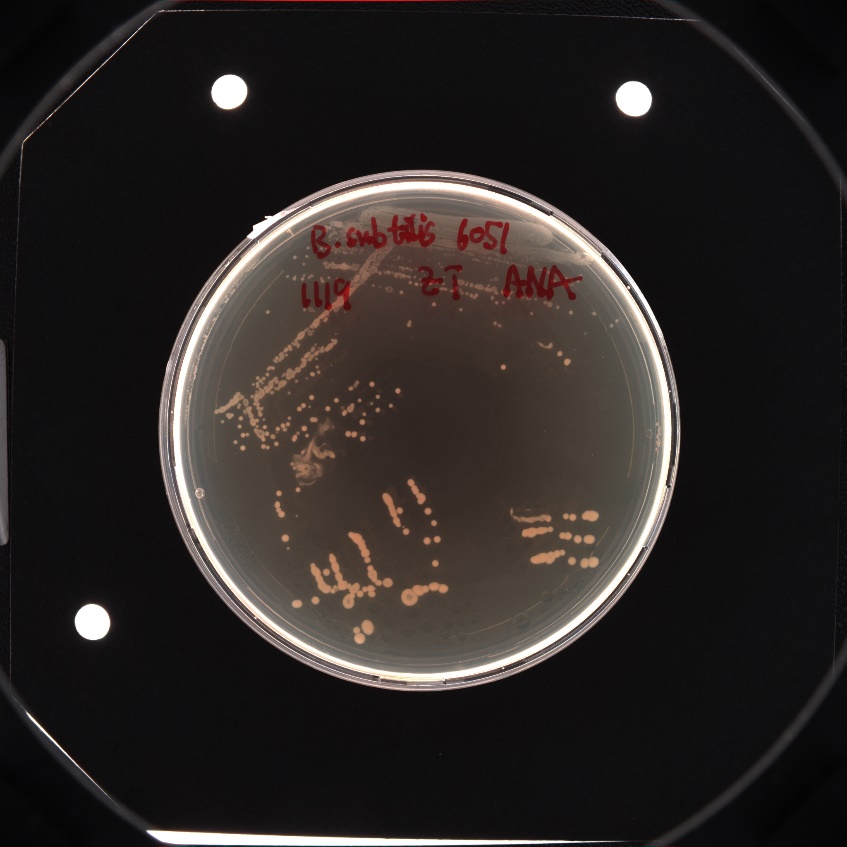


(c)
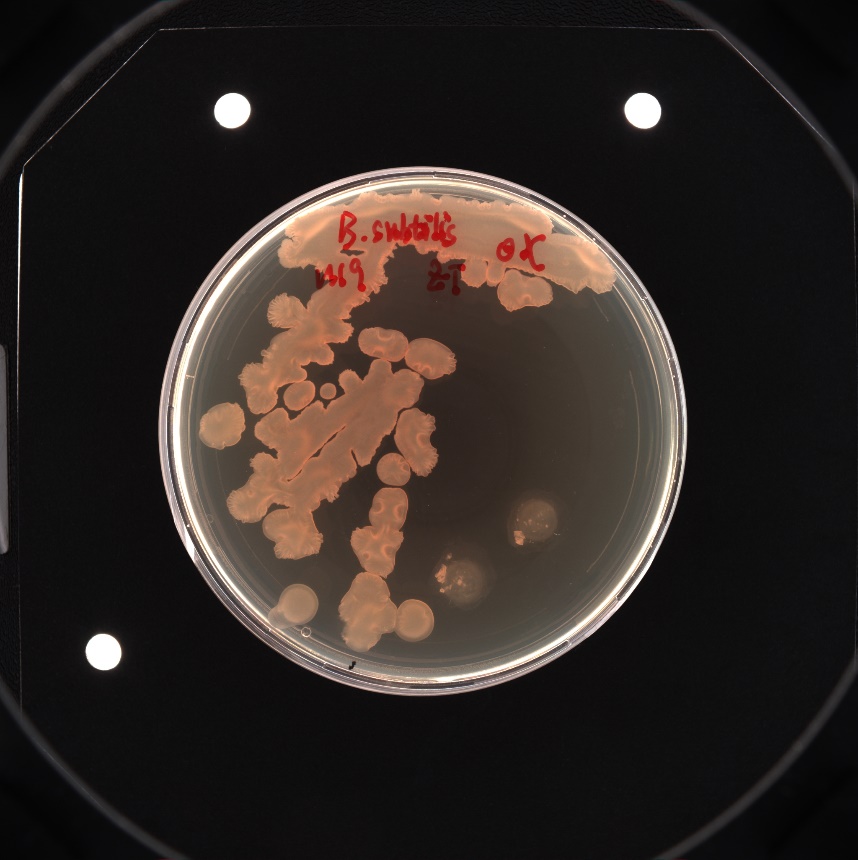
 (d)
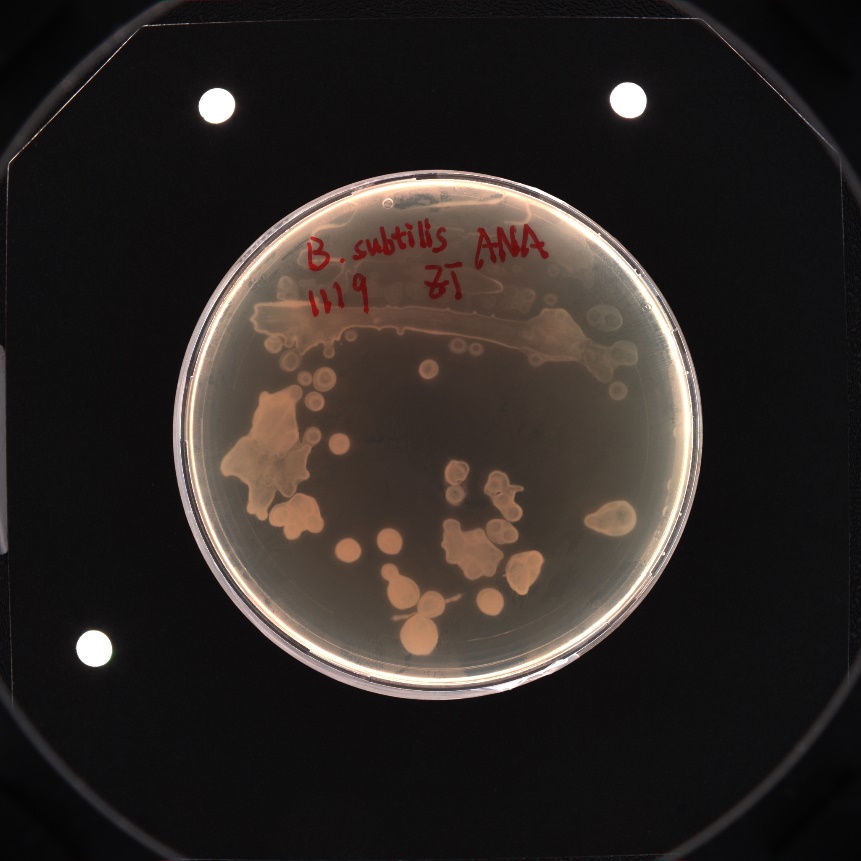


**Figure S6.** Growth of B. subtilis ATCC 6051 and B. subtilis SOM8 under aerobic and anaerobic conditions respectively. (a) B. subtilis ATCC 6051-OX; (b) B. subtilis ATCC 6051-ANA; (c) B. subtilis SOM8-OX; (d) B. subtilis SOM8-ANA.

**(a)**
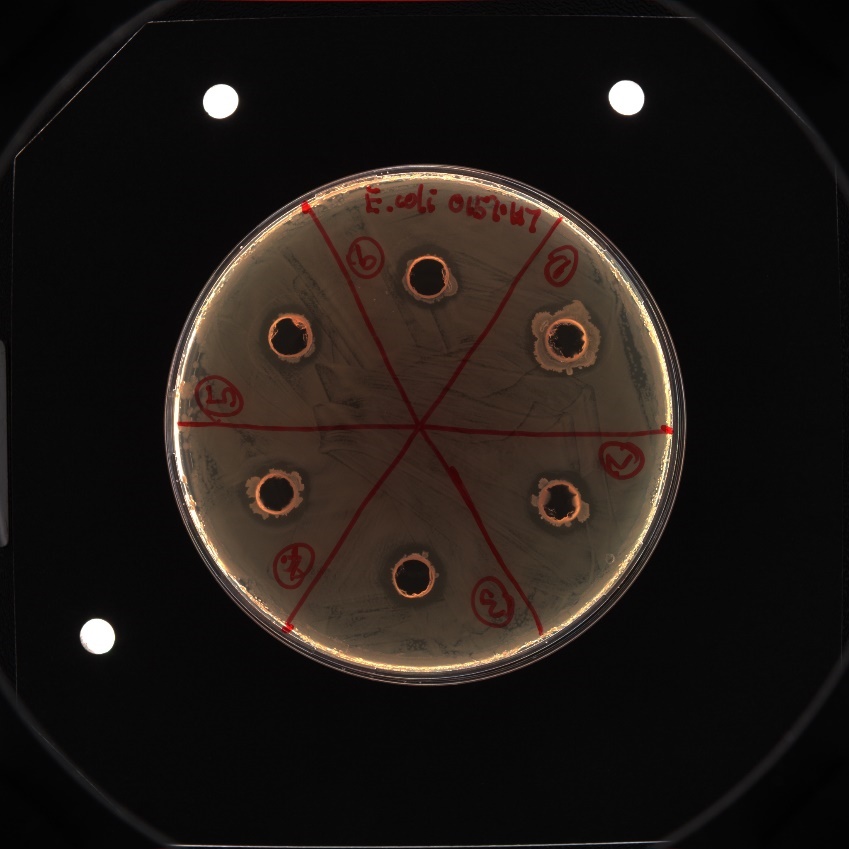
 **(b)**
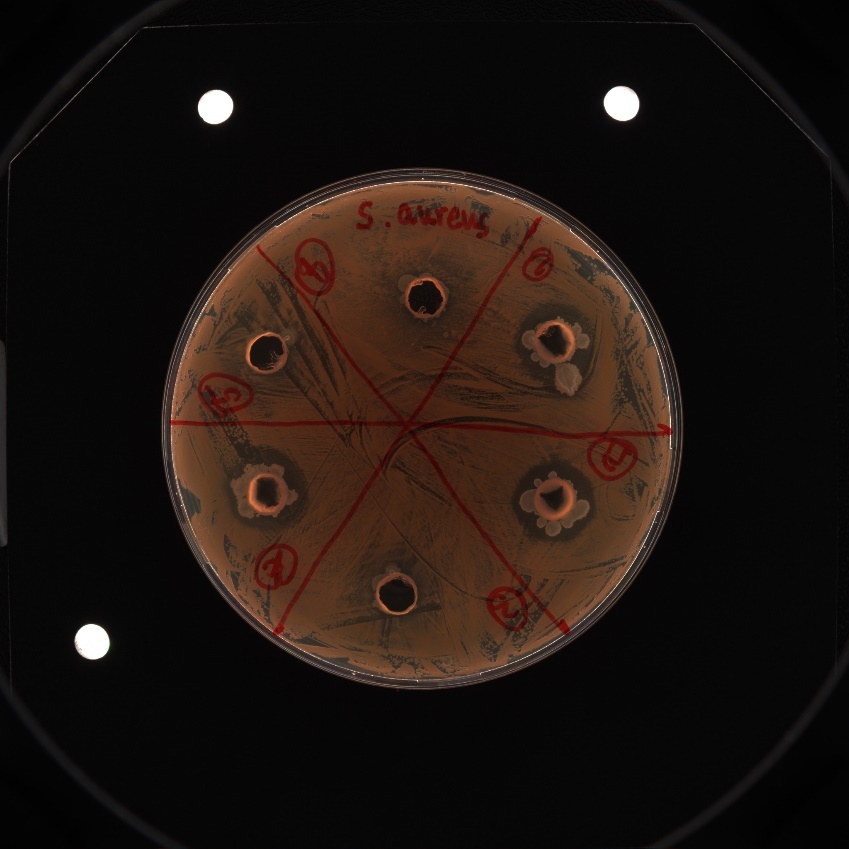


**(c)**
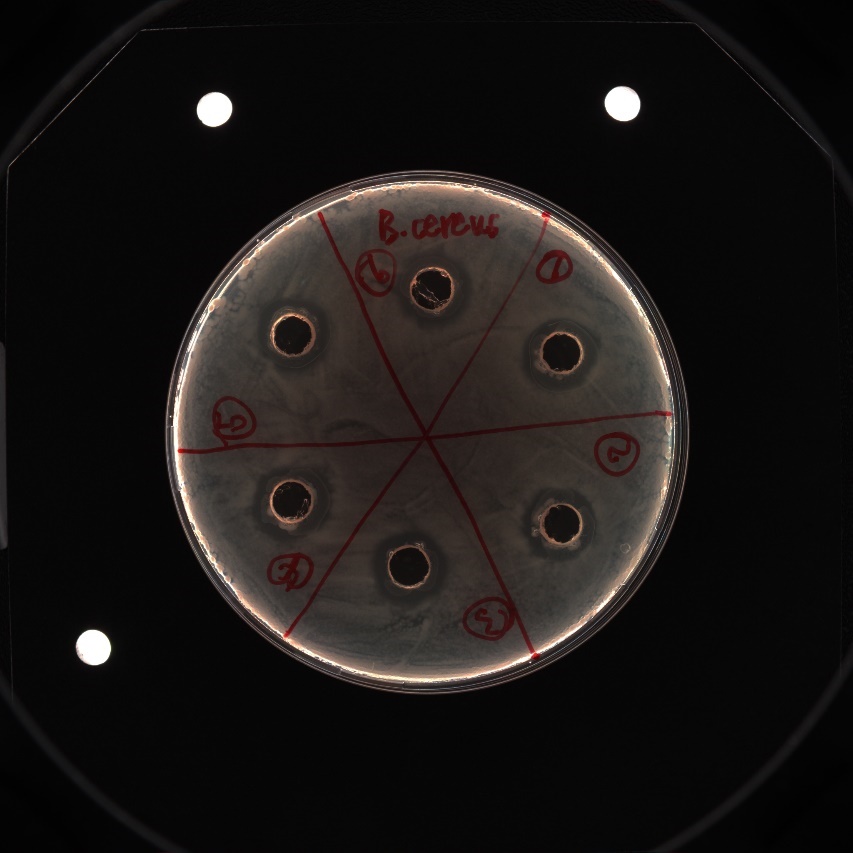
**(d)**
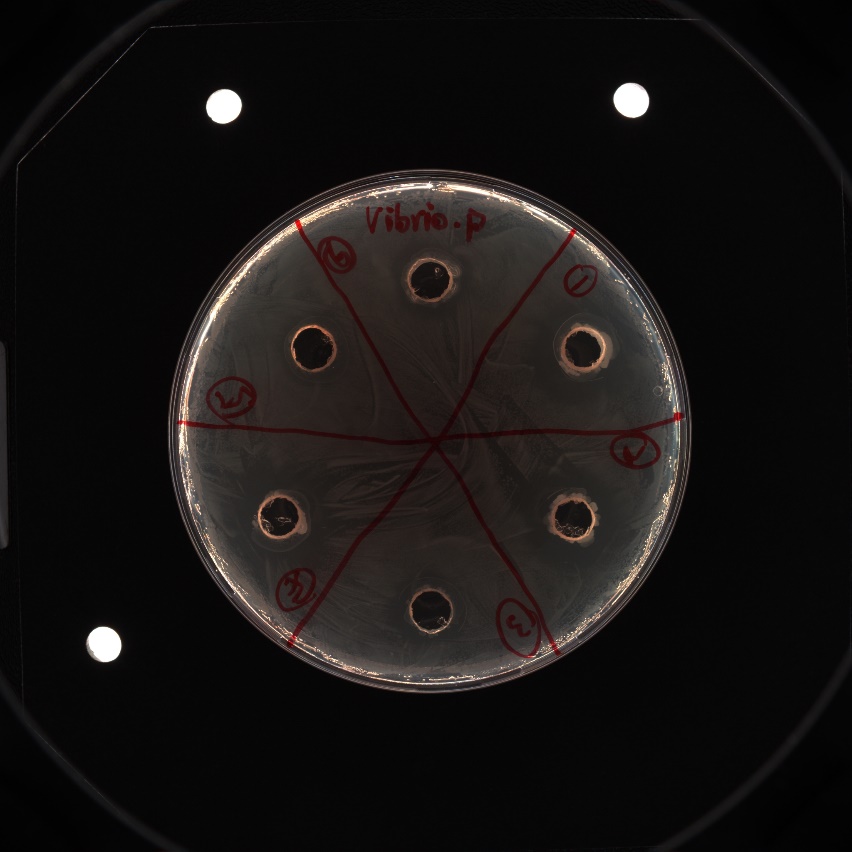


**Figure S7.** Examples of antimicrobial activities of B. subtilis SOM8 and B. subtilis ATCC 6051 against human enteropathogen. Number 1 to 6 are B. subtilis SOM8 (OX-OX: Firstly, grown in aerobic condition, then incubate aerobically), B. subtilis SOM8 (OX to ANA), B. subtilis SOM8 (ANA-ANA), B. subtilis ATCC 6051 (OX-OX), B. subtilis ATCC 6051 (OX to ANA), B. subtilis ATCC 6051 (ANA-ANA) respectively. (a) E. Coli O157:H7; (b) S. aureus; (c) B. cereus; (d) V. parahaemolyticus.
